# Neuro-Immune Hemostasis: Homeostasis and Diseases in the Central Nervous System

**DOI:** 10.3389/fncel.2018.00459

**Published:** 2018-11-26

**Authors:** Ciro De Luca, Anna Maria Colangelo, Lilia Alberghina, Michele Papa

**Affiliations:** ^1^Laboratory of Morphology of Neuronal Network, Department of Public Medicine, University of Campania-Luigi Vanvitelli, Naples, Italy; ^2^Laboratory of Neuroscience “R. Levi-Montalcini”, Department of Biotechnology and Biosciences, University of Milano-Bicocca, Milan, Italy; ^3^SYSBIO Centre of Systems Biology, University of Milano-Bicocca, Milan, Italy

**Keywords:** coagulation, complement, neuro-immune system, neuroinflammation, Alzheimer’s disease, vascular diseases, brain tumor, systems biology

## Abstract

Coagulation and the immune system interact in several physiological and pathological conditions, including tissue repair, host defense, and homeostatic maintenance. This network plays a key role in diseases of the central nervous system (CNS) by involving several cells (CNS resident cells, platelets, endothelium, and leukocytes) and molecular pathways (protease activity, complement factors, platelet granule content). Endothelial damage prompts platelet activation and the coagulation cascade as the first physiological step to support the rescue of damaged tissues, a flawed rescuing system ultimately producing neuroinflammation. Leukocytes, platelets, and endothelial cells are sensitive to the damage and indeed can release or respond to chemokines and cytokines (platelet factor 4, CXCL4, TNF, interleukins), and growth factors (including platelet-derived growth factor, vascular endothelial growth factor, and brain-derived neurotrophic factor) with platelet activation, change in capillary permeability, migration or differentiation of leukocytes. Thrombin, plasmin, activated complement factors and matrix metalloproteinase-1 (MMP-1), furthermore, activate intracellular transduction through complement or protease-activated receptors. Impairment of the neuro-immune hemostasis network induces acute or chronic CNS pathologies related to the neurovascular unit, either directly or by the systemic activation of its main steps. Neurons, glial cells (astrocytes and microglia) and the extracellular matrix play a crucial function in a “tetrapartite” synaptic model. Taking into account the neurovascular unit, in this review we thoroughly analyzed the influence of neuro-immune hemostasis on these five elements acting as a functional unit (“pentapartite” synapse) in the adaptive and maladaptive plasticity and discuss the relevance of these events in inflammatory, cerebrovascular, Alzheimer, neoplastic and psychiatric diseases. Finally, based on the solid reviewed data, we hypothesize a model of neuro-immune hemostatic network based on protein–protein interactions. In addition, we propose that, to better understand and favor the maintenance of adaptive plasticity, it would be useful to construct predictive molecular models, able to enlighten the regulating logic of the complex molecular network, which belongs to different cellular domains. A modeling approach would help to define how nodes of the network interact with basic cellular functions, such as mitochondrial metabolism, autophagy or apoptosis. It is expected that dynamic systems biology models might help to elucidate the fine structure of molecular events generated by blood coagulation and neuro-immune responses in several CNS diseases, thereby opening the way to more effective treatments.

## Introduction

The complex and dynamic biological process of healing following an injury is linked to the activation of coagulation and of the immune system (Mancuso and Santagostino, 2017; Wang et al., 2017). Indeed, reparative processes can functionally restore endothelial integrity after a damage (infectious, post-traumatic, shear stress-induced, or metabolic-related) and hereafter activate the immune system (Nurden, 2011; Mancuso and Santagostino, 2017). Activation of these integrated processes relies upon cellular components and circulating factors that are crucial to stimulate physiological repair and tissue rearrangement, and essential to the homeostasis of central nervous system (CNS) (De Luca et al., 2017).

The paramount step to secure the integrity of the system is the scouting of the endothelial damage and the clot formation. The coagulation players have been divided into cellular and protease components, the latter playing into three pathways: intrinsic, extrinsic, and common (McMichael, 2012). In this review the clotting factors will be addressed with Roman numbers, with the post-position of the letter “a” to indicate the activated factor (Giangrande, 2003) except for the FI-IV that will be addressed as fibrinogen, prothrombin, tissue factors (TFs) and Calcium (Ca^2+^) (Table 1).

**Table 1:** List of the coagulation factors with their assigned Roman numbers and the alternative names that are found in the literature.

Factor number	Alternative name(s)
I	Fibrinogen (fibrin zymogen)
II	Prothrombin (thrombin zymogen)
III	Tissue factor
IV	Calcium
V	Labile factor, proaccelerin
VI	Unassigned (previously activated factor V)
VII	Stable factor (proconvertin)
VIII	Antihemophilic factor A
IX	Christmas factor, antihemophilic factor B
X	Stuart prowar factor
XI	Plasma thromboplastin antecedent
XII	Hageman factor
XIII	Fibrin stabilizing factor

The explorers of damage are FVII (described as the extrinsic pathway initiator), the only human factor circulating as both FVII and FVIIa to monitor TF exposure, and FXII (the intrinsic pathway initiator), whose activation is induced by subendothelial collagen in the presence of high-molecular-weight kininogen (HMWK). The TF-FVIIa complex activates FIX and FX, while FXIIa activates FXI, which also leads to FIXa. FXa indeed converts low levels of prothrombin in thrombin (Tanaka et al., 2009). Briefly, low concentration of thrombin and FXa are the scouting signals that can either trigger the amplification or be neutralized by the surrounding healthy cells.

Platelet are then activated with the formation of pseudopodia that allow covering the injury site and the release of granular (α and δ granules) mediators (Mancuso and Santagostino, 2017). In fact, once the damage has been localized, degranulation of α-granules exposes P-selectin, activates integrin αIIbβ3, promotes circulating immune cells adhesion and releases fibrinogen, VWF, FV and more than 300 proteins, including chemokines (Bahou, 2013) and growth factors, such as platelet-derived growth factor (PDGF), brain-derived neurotrophic factor (BDNF), fibroblast growth factor (FGF), and the vascular endothelial growth factor (VEGF) (Nurden, 2011; Chen et al., 2018). P-selectin, also known as Cluster of Differentiation (CD)62P, and glycoprotein (GP)Ib on platelets are respectively recognized on white cells by P-selectin glycoprotein ligand 1 (PSGL1) and αMβ2 integrin CD11b/CD18, also known as Macrophage-1 antigen (Mac-1) or CR3, the latter able to bind also complement, fibrinogen and platelet GPIIb-IIIa (Nurden, 2011; Mancuso and Santagostino, 2017). Chemokines, or chemotactic cytokines, are classified into families according to the arrangement of the cysteine residues that constitutes the disulfide bridges, namely CXCL, CC, CX3C, XC (Zlotnik and Yoshie, 2000, 2012). Among chemokines, platelet factor 4 (PF4, also known as CXCL4), CXCL1, interleukin-8 (IL-8) (Bahou, 2013) and CXCL7 are absolutely the most abundant, the latter used as a marker of megakaryocytic lineage. However, several other molecules (CCL5, CXCL5, CXCL12) have been recognized, and there is evidence of distinct and specialized α-granules subclasses containing subsets of chemokines specifically released under certain pathophysiological conditions (Italiano et al., 2008; van Nispen tot Pannerden et al., 2010).

The δ granules (or dense granules) release bioactive amines (e.g., histamine and serotonin), adenine nucleotides (ADP, ATP, and cAMP), poly- and pyrophosphates, and high concentrations of cations, above all Ca^2+^ (Bahou, 2013). A high local concentration of FXa and thrombin ends the amplification phase and induces its propagation and the last proteolytic cleavage that involves fibrinogen, which is then transformed into fibrin and stabilized by the transglutaminase FXIIIa. Plasminogen, activated by tissue-type plasminogen activator (tPA) into the protease plasmin, can ultimately disassemble the fibrin aggregates (Muszbek et al., 2011). Moreover, thrombin on the intact vessel wall can inhibit itself: it binds to thrombomodulin and cleaves Protein C (PC) into its activated form (aPC) which, together with Protein S and TFPI, inactivates FVa, FVIIIa and inhibits FXa, thus limiting and quenching the process (Peraramelli et al., 2012; Mosnier et al., 2014).

Moreover, on cell surface thrombin can trigger the complement cascade (Hamad et al., 2010). Activated complement receptor (CR)2 is present on resting platelets expressing GPIb, as well as the iC3b binding on CR3, while C3a, C5a (the so-called anaphylatoxin) and C4a are bound on human platelets (Cosgrove et al., 1987). However, to inhibit spontaneous aggregation and membrane attack complex (MAC) formation, it is pivotal the expression of Factor H (FH) and thrombospondin type 1 (TSP-1) (Mnjoyan et al., 2008). The important regulation of this system has been demonstrated, for example, in the atypical hemolytic uremic syndrome (aHUS), where the deficit of FH (genetic or acquired) causes the triad: anemia, thrombocytopenia, and uremia (due to acute renal failure) (Kavanagh et al., 2013).

## Neuro-Immune Hemostasis and Neuroinflammation

The complexity of the neuro-immune system interaction starts at the level of the neurovascular unit. This structure is composed by the blood–brain barrier (BBB) elements (endothelial cells, pericytes and the astrocytic foot endings) on the one side, and neurons, glia and extracellular matrix (ECM) on the other side (Muoio et al., 2014). All together, these elements (neurons, glia, and ECM) constitute what has been well-described as the tetrapartite synapse (Dityatev and Rusakov, 2011; Tsilibary et al., 2014; De Luca and Papa, 2016, 2017).

This complex structure accounts for the immune privileged properties of the brain and of the spinal cord (which has its own blood–barrier BSCB) (Bartanusz et al., 2011). The efficacy of the barrier is based on the innate immune properties of resident cells (neurons and glia), which interact with pathogens, endogenous (e.g., unfolded or misfolded proteins) or exogenous toxins, to maximize the adaptive response and to reduce the collateral damage (Medzhitov et al., 2012; Lampron et al., 2013). The resiliency of the system strictly depends on a variety of proteins that have been grouped for their functional role of neuro-immune regulators (NIReg) and are expressed on both glial cells and neurons (Bedoui et al., 2018).

**FIGURE 1:**
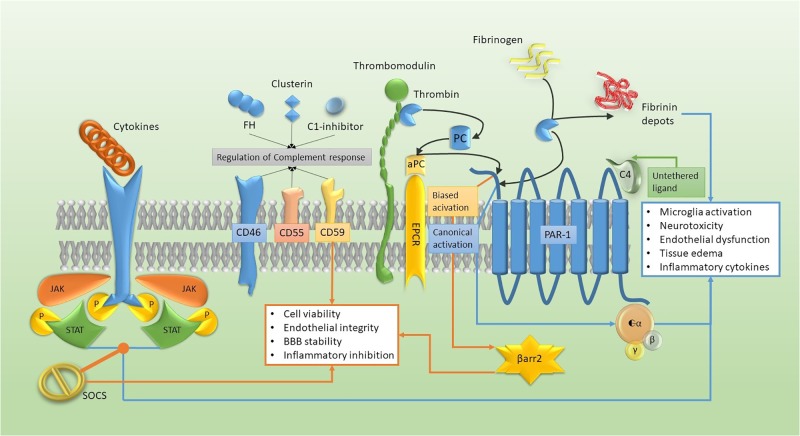
Molecular pathways of the neuro-immune hemostasis model. Cytokine activation of JAK-STAT tyrosine kinase receptor can be turned off by SOCS expression and, together with the complement regulation and the biased activation of PAR-1/β arrestin (β arr2) (orange pathway), they enhance the adaptive response. The canonical activation of PAR1, together with tyrosine kinase receptor activation and the fibrin deposition (blue pathway), accelerate maladaptive cellular changes.

**FIGURE 2:**
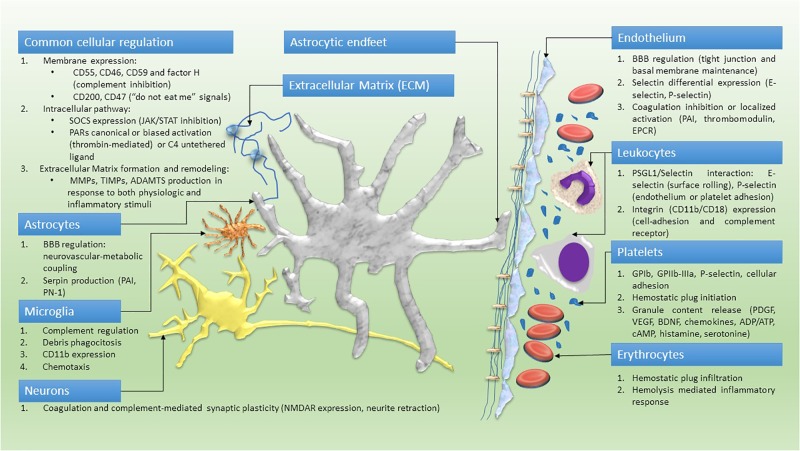
Cellular model of a pentapartite synapse. Common cellular pathways and cell-specific role within the neuro-immune hemostasis network.

NIReg are constitutively expressed on neurons and acts as “Don’t eat me” signals for microglial cells that are maintained in a resting state (such as the CD200, CD47, and CXCL1), or they negatively regulate complement activation (CD59, CD46, FH) (Bedoui et al., 2018) (Figures 1, 2). Moreover, resident cells are able to repress deregulated cytokine activation through the expression of Suppressor Of Cytokine Signaling (SOCS) that interferes with the inflammatory-related activation of the JAnus-Kinase (JAK)/Signal Transducer and Activator of Transcription (STAT) intracellular polarizing pathway (Bedoui et al., 2018).

Thrombin, a crucial protein in this network, has been reported to affect the behavior of CNS resident cells, such as neurons (Olianas et al., 2007), astrocytes and microglia (Lin et al., 2013; Beggs and Salter, 2016) in a dose dependent manner, with adaptive changes at low dose and maladaptive modifications at high concentration.

Of relevance for thrombin interaction with CNS resident cells, endothelium, and immune cells, is the proteinase activated receptor (PAR) family, G protein coupled receptors (GPCR)s with four recognized members (PAR1-4). PARs can be activated by a proteolytic cleavage of the extracellular amino-terminal domain of their seven-transmembrane α-helix structure (Junge et al., 2004; Wang et al., 2004; Luo et al., 2007; Traynelis and Trejo, 2007; Vance et al., 2015). Thrombin (not complexed with thrombomodulin), FXa, plasmin, and matrix metalloproteinases-1 (MMP-1) canonically activate PAR1 on cell surface, unmasking the tethered ligand with a proteolytic cleavage and causing its internalization (Ye et al., 1994; Wittinghofer and Vetter, 2011). Thrombin can alter neuronal viability through PAR-1 interaction, since thrombin antagonists and pharmacological/genetic PAR-1 inhibitors increase neuroprotection (Rajput et al., 2014). PAR2, instead, can be activated by low-concentration of trypsin. PAR3 is the only PAR with no function because of its short cytoplasmic domain (Coughlin, 2005). Indeed, it can act as a cofactor for other PARs to form heterodimers and amplify the variety of intracellular transduction systems (Arachiche et al., 2013; Lin and Trejo, 2013), perhaps by allosteric modification and interaction with different G proteins (Gαq/11, Gαi/o, Gα12/13) (Ossovskaya and Bunnett, 2004; McLaughlin et al., 2005). Non-canonical cleavage by aPC also activates PAR1 (biased agonism) and trigger a different intracellular pathway (Figure 1). The aPC binds to the coreceptor endothelial PC receptor (EPCR) and cleaves PAR1 (Riewald and Ruf, 2005), but does not induce its internalization as being co-localized in caveolin-rich membrane domains (Russo et al., 2009). Canonical-cleavage activates Guanosine-triphosphatase (GTPase) rat-sarcoma protein (Ras)-related protein-A (RhoA), while non-canonical cleavage (aPC-mediated) stimulates GTPase Ras-related C3-botulinum toxin substrate-1 (Rac1) through β arrestin (βarr2) and disheveled 2 (Dvl-2), usually associated to frizzled receptors (FZD) (Russo et al., 2009; Soh and Trejo, 2011). Moreover, C4a has been proposed as an untethered agonist for PAR1 and PAR4 acting through Gαq-Phospholipase C (PLC) pathway that within nanomolar concentrations can cause intracellular Ca^2+^ release (Wang et al., 2017) (Figure 1).

NIReg also include serpins and thrombomodulin, which are able to reduce the potential neurotoxicity of thrombin, mainly mediated through tethered ligand activation of PAR1 signaling (Buisson et al., 1998; Niego et al., 2011) (Figure 1). The lipopolysaccharide (LPS), furthermore, stimulates the expression of coagulation and inflammation factors in the hippocampal microglia, but a sharp reduction of both inflammatory and coagulation factors occurs when the thrombin-mediated signal is inhibited (Shavit Stein et al., 2018).

Definitely, based on relevance of the neurovascular unit in modulating neuro-immune hemostasis and neuroinflammation, we propose that the neurovascular complex can be associated with the tetrapartite synapse to form an ultimately unified pentapartite synaptic model (Figure 2). This model might better represent the complex and robust interplay between cellular and molecular pathways of coagulation and the immune system, as reported by *in vitro* or *in vivo* studies (Thornton et al., 2010; Barbier et al., 2011). The analysis of the state of the art in this field can partly reveal the pathophysiology of neuro-inflammatory and neurodegenerative diseases, such as multiple sclerosis (MS), cerebrovascular, Alzheimer, neoplastic and psychiatric diseases.

## Multiple Sclerosis

Multiple sclerosis is a demyelinating autoimmune inflammatory disease affecting the CNS white matter. It lacks a commonly recognized causative agent (idiopathic), and the multifactorial interactions between environment and genetics are not fully elucidated (Sawcer et al., 2014; Belbasis et al., 2015). Though the pathophysiology of MS remains unknown, there is morphological evidence of its inflammatory origin and of the resulting neurodegeneration, moreover, therapies targeting the inflammasome modify the progression of the disease (mainly the relapsing-remitting phenotype) (Dahdaleh et al., 2017).

On the base of the clinical observation and the progression, MS can be classified into two forms, relapsing-remitting and progressive (primary or secondary) (Lublin and Reingold, 1996). Inflammation with relatively preserved cell viability seems to be the hallmark of relapsing-remitting early stages, is characterized by clinical features that can affect the motor system (particularly the pyramidal tract) or non-motor areas, depending on which part of the CNS is affected by the demyelination. Every relapse is followed by a spontaneous partial remission, ameliorated by early therapy (Lublin and Reingold, 1996), while the progressive forms, either the primary or the evolution of the initially relapsing-remitting MS (secondary), are characterized by a continuous neurodegeneration with almost ineffective therapy on its progression (Lublin and Reingold, 1996; Feinstein et al., 2015).

Which is the key to understand the failure of the immune system has been long debated. Inflammatory autoimmunity, defined “horror autotoxicus” by Paul Ehrlich over a century ago (Ehrlich, 1900), starts with the erroneous recognition of an endogenous target as a threat, with the activation of resident cells that present it to the immunity effectors. As discussed above, the neurovascular unit should prevent inappropriate migration of leukocytes from the bloodstream and protect the CNS. The Trojan horse that could cause the BBB failure and allow the specific T-cells diapedesis has not been identified yet, but a putative role could be assigned to platelets activation and fibrin depots in the CNS and other tissues (Hultman et al., 2014; Joshi et al., 2016). These cellular and protein aggregates can be produced by a minimal vascular damage or venous stasis, and their pathological accumulation could produce a non-diffusible and localized signal to mediate lymphocyte T helper (Th)1 migration and myelin targeting (Ryu et al., 2015). This hypothesis is supported by the evidence of the occurrence of fibrinogen in myelinated areas that correlates with T-cells invasion and IL-12 mediated Th1 differentiation, macrophage activation through CCL2 and CXCL10 and following demyelination (Lodygin et al., 2013). Antibodies directed to GPIb or GPIIb-IIIa reduce the severity of the disease in an animal model, whereas increased integrin αIIb gene (ITGA2B) mRNA has been found in chronic lesions of MS patients (Lock et al., 2002; Langer et al., 2012). Furthermore, in the autoimmune encephalomyelitis model (EAE) it has been shown that depletion of fibrinogen blocks T-cell activation, accelerating the coagulopathy-mediated pathway (Akassoglou et al., 2004), where the presence of fibrin or platelets are the erroneous triggering signal, allowing antigen presenting cells to interact with lymphocytes and delete the immune privilege property of the CNS (Lodygin et al., 2013). Thrombin, in addition, can speed the process between platelet activation and fibrin deposition (Figure 1) favoring the formation of aggregates, and drive the leukocytes through release of granular CCL3, CCL5, PF4 (CXCL4), and CXCL5 (Raphael et al., 2015).

Platelet activation is associated with clinically assessed worsening of MS, as demonstrated by the positive correlation between the Expanded Disability Status Scale (EDSS) and the level of Cyclooxygenase (COX)-1 activity, and the levels of the eicosanoid thromboxane B2 (TBX2) in blood platelets of MS patients (Morel et al., 2016). Furthermore, mediators from platelet granules include growth factors (i.e., BDNF) and chemokines involved, together with MMPs, in both hemostasis and inflammatory progression (Morrell et al., 2014). Hence, the initial vascular damage activates platelets and converts fibrinogen. Platelets phenotype, at this point, is characterized by low selectin expression and low-rate degranulation. The granule content (PF4, serotonin, chemokines, cytokines, ADP, ATP, etc.) is released more efficiently upon contact with astrocytes, neuronal glycolipids and ECM (Sotnikov et al., 2013) (Figure 2). The acute inflammatory response is not produced only by the active degranulation; the expression of other proteins is needed to activate the inflammasome. One of the main proteins involved, and utterly the most studied, is Interleukin-1β (IL-1β) synthesized on demand from a messenger RNA stored inside platelets. IL-1β is secreted and found in occlusive thrombi after vascular damage (before leukocyte incorporation) (Mancuso and Santagostino, 2017). Moreover, the corresponding receptor (IL1R) is expressed on the platelet surface, allowing an autocrine self-sustaining feedback (Brown et al., 2013). Tumor necrosis factor-α (TNF-α) also seems to be influenced by platelets activation, since its depletion significantly reduces white cells diapedesis during TNFα-mediated CNS phlogosis (Carvalho-Tavares et al., 2000). TNF-α can induce cellular apoptosis or contribute to chemokine generation and neurodegeneration through necroptosis (Dhuriya and Sharma, 2018).

This unregulated process induces BBB leakage, antigen presentation, activation of CD4 positive T-cells and their differentiation into Th1 or Th17 (Starossom et al., 2015). During later stages, the process is self-sustaining and platelets change their phenotype, express a higher level of selectins and adhere to T-cells or to antigen presenting cells, forming aggregates (Starossom et al., 2015).

The pivotal role of the molecular components of the coagulation cascade is confirmed by the protective action in the EAE model exerted by the administration of both warfarin and the non-vitamin K antagonist oral anticoagulant (NOAC) rivaroxaban (direct inhibitor of FXa) (Stolz et al., 2017). Thrombin can interrupt BBB integrity through PAR1 signaling; however, warfarin effect seems to be greater than rivaroxaban, probably resulting for the interactions with other proteins of the coagulation pathway, such as FVII (Coughlin, 2000, 2005). The lysis of fibrin, furthermore, should inhibit microglia activation via CD11b (part of CR3, encoded by ITGAM gene) and produce a similar beneficial effect in the EAE model without the possible detrimental side effect of warfarin due to the inhibition of the aPC endothelial protective functions (Adams et al., 2007; Sarangi et al., 2010). The protective role of aPC, however, is not a linear process; it has been shown that the anticoagulant and the intracellular signaling function of aPC are both essential to exert neuroprotection. However, both the inhibition of the endogenous aPC and the administration of exogenous aPC ameliorated the EAE outcome (Alabanza et al., 2013). This seems apparently controversial, but it can be explained because reduced endogenous aPC can induce early leukocyte infiltration due to BBB instability; at the same time, it increases CD11b positive elements, interfering with T-cells differentiation, thus reducing disease progression (Alabanza et al., 2013). Reduction of aPC is effective only if it is induced before the disease onset (Alabanza et al., 2013).

As the aforementioned CD11b positive elements confirm, the complement system pathway is a prominent component of the immune response and is entirely involved in inflammatory autoimmune disease at various levels. The system plays a pivotal role in essentially all immune-mediated processes, and it made difficult, although intriguing, designing drugs targeting its components. Complement proteins can act through three main activation pathway: classical, lectin and alternative pathway, all at the end forming the MAC complex with the elimination of target cells (Horstman et al., 2011). A coding variant in the C3 gene (C3R102G) reduced C3 activity and affected white/gray matter damage that is related to increased cognitive dysfunction in MS patients (Roostaei et al., 2018). Detrimental effects of complement interference can be overcome through inhibition at the lower level of complement activation cascade (e.g., C5). This strategy may reduce detrimental inflammation without affecting previous signaling steps. Hence, Eculizumab an inhibitor of C5, already approved for treatment of the paroxysmal—nocturnal hemoglobinuria (PNH), and of the aHUS (Marti-Carvajal et al., 2014; Cofiell et al., 2015) is undergoing phase III clinical trial for another autoimmune demyelinating disease of the CNS, the neuromyelitis optica (NMO) either for prevention or relapses forms^1^. NMO with dominant involvement of optic nerves and spinal cord has been previously considered as a variant of MS. Nowadays, it is considered a distinct disease (Trebst et al., 2014) and even though it can lead to a more early disability, there are few approved therapeutic protocols (Trebst et al., 2014). Furthermore, human post-mortem study has demonstrated that neurons from patients with progressive MS express a higher level of PAR1 and are exposed to granzyme B and IL-1β, inflammatory mediators that could at least partially explain by their neurotoxic effect the progressive related cortical atrophy (Lee et al., 2017).

## Cerebrovascular Diseases

The link between coagulation and cerebrovascular diseases seems causative, however, recent findings confirm the pivotal role of the coagulation/immune pathways during the plastic remodeling following or associated to the acute event. After an ischemic and hemorrhagic stroke, cortical synaptic remodeling is largely biased by a maladaptive response accounting for about 10% of epileptic seizures in elder (Maggio et al., 2008; Procaccianti et al., 2012; Guth et al., 2014). Moreover, the efficacy of thrombolysis and the current guidelines for the management of acute ischemic stroke (AIS) have rapidly reduced the acute mortality rate. Nowadays, the main social burdens and future challenges concern prolonged hospitalization, ineffective rehabilitation program, disability, and the epileptic sequelae (Szaflarski et al., 2008; Burneo et al., 2010; Tsivgoulis et al., 2014; GBD, 2016). Studies on human platelet activation focused on platelet ultrastructure and plasma levels of PF4, thrombin–antithrombin complex, fibrinogen and other biomarkers in patients with AIS, and during the chronic phase or with atherosclerosis (without stroke) compared to healthy subjects (Kurabayashi et al., 2000). Data are compatible with platelet pre-activation in atherosclerotic patients, similar but lower compared to patients with AIS or chronic phase. Pre-activated platelets showed a higher density of pseudopods and vacuoles (Kurabayashi et al., 2000, 2010). The endothelial damage reported in atherosclerosis, combined with a pre-activated state of platelets, is the most plausible factor for inflammatory-mediated CNS damage that accelerates the major hypoxic injury.

Astrocytes and other glial cells play a key role in the rearrangement of the neurovascular unit. They react to inflammatory stimuli by expressing chemokines (CXC and CC type) (García-Berrocoso et al., 2014). The CCL20, expressed only by astrocytic cells, is essential for the recruitment of white cells to the CNS (Ambrosini et al., 2003). The concentration of CCL22 is reduced in the damaged brain tissue and in the bloodstream following AIS and is associated with poor stroke outcome (García-Berrocoso et al., 2014). The CCL22 receptor CCR4, expressed on Th2 cells can shift the inflammatory response toward an adaptive deregulation, suppressing Th1-polarized response. This is supportive for a protective immune response, initiated during the acute ischemic event and lasting for some time (Theodorou et al., 2008). Microglia express C1q during transient ischemic attack and deposits of C3d and C9 have been reported on neuronal cells after traumatic concussion (Pasinetti et al., 1992; Schafer et al., 2000; Schultz et al., 2005). Depletion of the complement factor, C1q deficiency, and C1-inhibitor expression (Figure 1) are neuroprotective too, improving blood flow and neurological recovery after transient ischemia, and reducing the intracerebral edema following hemorrhage (Vasthare et al., 1998; Xi et al., 2001; Ten et al., 2005).

According to these data, complement levels in the cerebrospinal fluid (CSF) are elevated following AIS and correlate with BBB failure. C1q, C3b, and C3d have been found close to the ischemic penumbra (Lindsberg et al., 1996; Pedersen et al., 2004; Schultz et al., 2005). All these data show the putative role of complement activation in tissue recovering after AIS, thus offering new perspectives on the role of complement during the postictal plastic reorganization.

Thrombin effects depend on its concentration: the increase produces an adaptive-maladaptive gradient (Maggio et al., 2013c; Becker et al., 2014; Ben Shimon et al., 2015; Bushi et al., 2015; Garcia et al., 2015). Following AIS in the surrounding non-ischemic tissue, thrombin concentration is high; this could influences the recovery in the ischemic penumbra. In the PAR1 null animal model, a reduction of the ischemic area and repair in the postictal phase has been reported (Hamill et al., 2009). Drug inhibition of PAR1, or its knockdown, restores the impaired synaptic plasticity and neurotransmission in hippocampal slices exposed to oxygen and glucose deprivation (OGD) (Stein et al., 2015). Thrombin is produced in the brain and its concentration alters the homeostatic behavior in both pathology and physiology (Ben Shimon et al., 2015). In OGD and transient medial cerebral artery occlusion (TMCAO) models, the levels of endogenous thrombin and FX are elevated; this affects synaptic function and alters the coagulation process (Bushi et al., 2015; Lenz et al., 2015; Stein et al., 2015). Thrombin in the perilesional area can be derived by the brain tissue itself or by the bloodstream following to BBB failure. However, how endogenous thrombin is involved in the AIS or ischemic chronic phase remains not completely understood.

One hypothesis is that impairment of inhibitory neurotransmission by γ-aminobutyric acid (GABA), or by increased paired-pulse facilitation (PPF) also known as neural facilitation, would partially account for post-ischemic epileptic seizure (Maggio et al., 2008, 2013a,b; Ben Shimon et al., 2015). PPF is reduced by the administration of PAR1 antagonist or diazepam (GABAergic drug) (Bushi et al., 2015). PAR1 receptor sensitivity to different G proteins activation in different brain areas (or PAR1 partial activation) could be a good premise to explain the thrombin-dose related effects (McCoy et al., 2012; Maggio et al., 2013b). Hence, thrombin should be considered at least one of the triggers of the stroke-dependent seizures. This is a real perspective to pursue a therapeutical PAR1 inhibitory approach to treat epilepsy, not necessarily related to stroke. In the paradigm of PAR1 partial activation, a potential target is aPC; it efficiently interferes with inflammation, by promoting vascular integrity and sustaining the neuronal cells viability, thus improving the post-ischemic outcome (Cheng et al., 2003). We previously reported that BBB stability and endothelium integrity are mediated by aPC and PAR1 pathway (De Luca et al., 2017); furthermore, the aPC improves synaptic plasticity during rehabilitation, probably through EPCR/PAR1/Sphingosine1-phosphate receptor1 (S1P1R) intracellular activation (Maggio et al., 2014). S1P1R modulation with FTY720 (Fingolimod) receptor agonist, or with a novel more specific receptor agonist (RP101075) have shown to improve the clinical outcome of AIS and intracerebral hemorrhage (ICH) in animal models (Liu et al., 2013; Sun et al., 2016). Fingolimod, being approved as treatment for MS (for its immunosuppressive properties) reduce the capability of lymphocyte infiltration of CNS, reduce perilesional edema, preserve the neurovascular unit and polarize microglia toward the attenuated neuroinflammatory polarization through the STAT3 pathway (Sun et al., 2016; Qin et al., 2017).

A recent phase 2 clinical trial with Fingolimod in AIS has been completed^2^and another trial combinating Fingolimod with Alteplase bridging with Mechanical Thrombectomy in Acute Ischemic Stroke (FAMTAIS) is ongoing^3^. Whether this mechanism involves neurons or astrocytes PAR response is still debated, however, the co-localization of EPCR and PAR1 in specific brain areas (as hypothesized), mainly in the hippocampus, has been shown (Maggio et al., 2014). These reported data suggest that developing S1P1R agonists and thrombin inhibitors acting on PAR1 specific signaling, combined with the progress of thrombolytic therapies (or mechanical thrombectomy), will reduce both AIS lethality and post-ischemic hospitalization. Indeed, it is compelling to define the pathophysiology underlying the reparative processes of ischemic tissues and the perilesional area, having special attention to the neuro-immune coagulation pathways.

## Alzheimer’s Disease

Alzheimer’s disease (AD) is a degenerative, gradually progressive, irreversible form of cognitive degeneration, recognized as the most diffused type of dementia (Reiman, 2014), and responsible for up to 60–80% of an estimated population (in 2010) of 35.6 million people worldwide affected by cognitive decay (Reitz et al., 2011; Mayeux and Stern, 2012). Gaetano Perusini and Alois Alzheimer firstly described AD at the beginning of the 20th century (Macchi et al., 1997) with their studies on AD anatomopathological hallmarks represented by amyloid-β (Aβ) plaques and neurofibrillary tangles (aggregates of hyperphosphorylated tau protein), cortical atrophy and gliosis (Papa et al., 2014; Reiman, 2014). The classical phenotype of AD is called hippocampal type and is characterized by progressive and disabling loss of memory function, with an amnestic syndrome selectively compromising episodic memory during early stages (Dubois et al., 2014). AD variant phenotypes include diseases affecting primary cognitive function other than memory, with apraxia, loss of visual identification or aphasia (respectively biparietal, occipitotemporal, or logopenic variant) (Dubois et al., 2014). Advanced neuroimaging or biomarkers (mostly in the CSF) based on Aβ or tau proteins and genetic profiling searching for causative mutations of presenilin1/2 or amyloid-precursor protein (APP) and risk factors such as apolipoprotein-E ε4 allelic variant now facilitate clinical diagnosis (Dubois et al., 2014). We need to find, as Perusini speculated, “[…] a noxious agent, which causes the whole disease, also acts on the blood vessels or equally damages both the neuron and the blood vessels” (Macchi et al., 1997).

Platelets are pivotal and pleiotropic in the regulation of inflammation and repair processes. In AD they might play a role in the proteolytic processing of APP by carrying a disintegrin and metalloproteinase (ADAM)17 (Skovronsky et al., 2001; Evin et al., 2003) or by favoring the complement cascade initiation. ADAM family proteins are transmembrane proteases that can perform the so-called α-cleavage on APP (Siegenthaler et al., 2016). A-cleavage prevents Aβ formation by inhibiting APP sequential cleavage by an aspartyl protease called BACE1 (β site APP cleaving enzyme 1) and by the γ-secretase with the following release of amyloid peptides of different lengths and aptitude for plaque formation (Aβ-38, Aβ-40, Aβ-42) (Siegenthaler et al., 2016). The link between AD and complement proteins has been proposed after the deposition of C proteins was shown inside Aβ plaques. Subsequently, it was reported that amyloid itself could activate the complement cascade, even without the involvement of antibodies (as pattern recognition of damaged-self) (Salminen et al., 2009). This mechanism, overcoming the NIReg protective function, is functional in the general circulation, mediated by C3b and CR1 on erythrocytes. Furthermore, the genetic association between complement receptors or regulators (CR1 and clusterin) and AD has been suggested, opening the more vast scenario involving inflammatory regulators, lipid metabolism, complement, and platelets in the development of the disease (Jun et al., 2010). It has been demonstrated a coordinated involvement of clusterin, involved in complement-mediated cell lysis (also known as apolipoprotein j) (Figure 1), phospholipase-A2 (PLA2) and platelet-activating factor (PAF) (Osborn et al., 2007; Jun et al., 2010; Sanchez-Mejia and Mucke, 2010). The deficit of the early stage complement factors (e.g., C3) in AD mouse models has been shown to increase deposits of Aβ and accelerate the disease progression, probably due to a neuroprotective role played by the complement in clearing the plaque in the early phases of their formation (Wyss-Coray et al., 2002; Maier et al., 2008). These data must be considered with a caveat since the AD transgenic mouse model has been considered deficient in fully mimicking the human AD pathophysiology (Schwab et al., 2010). However, the immune system, considering the toll-like receptors (TLRs) or nucleotide-binding oligomerization domain (NOD)-like receptors and cytokines release, seems to share a common network with immune cells, complement and coagulation cascade through the damage-associated molecular patterns (DAMPs) recognition (Salminen et al., 2009).

Thrombin and the DAMP high-mobility group box protein 1 (HMGB1) have been associated to impaired memory formation acting cooperatively on the neuro-immune pathway, disrupting the neurovascular unit (Festoff et al., 2016). HMGB1 in particular seems to act via TLR4 and the receptor for advanced glycation end product (RAGE) impairing memory in an animal model (Mazarati et al., 2011).

Chronic vasculopathies and hypoxic damage have been reported in AD patient, along with fibrin depots in both capillaries and large vessels without the typical ischemic stepladder progression of the vascular dementia (Mazza et al., 2011). Besides the vascular hypo-perfusion and/or blood stasis, the fibrin deposition could play a signaling role in sustaining CNS inflammation and BBB failure, along with tight-junction digestion and ECM remodeling following blood cell invasion (Mosesson, 2005).

Considering Aβ deposition as a putative mechanism triggering the self-generating, self-sustaining degenerative process, and a DAMP itself prompting the immune system, Aβ-mediated expression of MMP-9 could alter the neurovascular unit and partially account for the intracerebral amyloid angiopathy (CAA) and the spontaneous parenchymal hemorrhages that almost constantly accompany disease progression (Hartz et al., 2012). Thrombin and FXIIIa are constituents of Aβ deposits in the vessel walls in CAA, suggesting a local activation mediated by amyloid, while FXIIIa can also form complexes with Aβ (both Aβ-40 and Aβ-42 fragments) by a mechanism that is independent of the binding site employed during fibrin ligation, and Ca^2+^ dependent (de Jager et al., 2015).

High thrombin concentration has a neurotoxic effect on microvessels of AD patient and can induce astrocytes apoptosis and BBB disruption followed by edema and blood leakage (Thal et al., 2008; Hartz et al., 2012; Schneider, 2016). Aβ-42 itself can be considered as a thrombin activating factor through FXI and FXII sequential activation, as shown in response to Aβ-42 oligomers (Zamolodchikov et al., 2016). This process further involves C1 inhibitor that can block FXIa, FXIIa, and kallicrein, endorsing the integration of the neuro-immune hemostatic model (Zamolodchikov et al., 2016). This thrombotic effect could be counterbalanced by soluble (s)APP expressing several Kunitz-type protease inhibitory domains, being able to block the protease functionality of coagulation factors at multiple levels (Ben Khalifa et al., 2012). The sAPP is produced during the Aβ-42 formation with a substantial possible balance between activation/repression of hemostasis. However, accumulation of one of these two fragments in several areas of CNS and blood vessels can account for the spotty aspects of thrombosis and hemorrhage, thoroughly described in the pathophysiology of the AD (Ramanathan et al., 2015). Protease nexin 1 (PN1) is a potent thrombin inhibitor released by glial cells that has been found extremely reduced and complexed with thrombin in AD patients, although its activity in Aβ depots was increased, probably due to the excess of thrombin (Vaughan et al., 1994; Baloyannis, 2015). Thrombin effects in AD, however, seem to be essentially regulated by PAR-1 through Gi/phosphatidylinositol-3 kinase (PI3K) signaling (Voss et al., 2007) and MMP-9 upregulation to prompt pathological effects on cells, endothelium, and ECM (Lin et al., 2014). Furthermore, MMP-9 activity is induced by plasmin, which converts pro-MMP-9 to active MMP-9. In addition to its effect on other ECM components, MMP-9 is involved in the degradation of nerve growth factor (NGF), a neurotrophin essential for development and function of cholinergic and other NGF-responsive neurons (Cuello et al., 2009; Cirillo et al., 2016). On the other hand, the tPA-plasmin system itself regulates the processing and maturation of pro-NGF and pro-BDNF to their corresponding mature forms (Lee et al., 2001; Pang et al., 2004; Bruno and Cuello, 2006; Colangelo et al., 2012). This implies that, in addition to the transcriptional events regulating neurotrophin levels (Colangelo et al., 2004), both thrombin and the tPA-plasmin system play a pivotal role in modulating neuronal differentiation and the structural changes linked to activity-dependent plasticity or Aβ toxicity.

## Neoplastic Diseases

Neoplasm developing inside the skull may be subdivided in primary lesions developing from resident cell clones (astrocytes, meninges, pituitary gland, bone or vascular structures, neurons or embryonic remnants) and secondary brain tumors, originating from systemic tumor metastasis, which are the most common (de Robles et al., 2015). The primary lesions from lung, breast cancers account for the 75% of metastasis, while melanoma, testicular and renal carcinomas are rare, but most likely tend to metastasize to the brain (Gallego Perez-Larraya and Hildebrand, 2014). The overall incidence of all brain tumors worldwide is 25.48 cases per 100,000 person (de Robles et al., 2015).

Several reports have shown a role of activated platelets in inflammatory chronic processes and metastatic cancer (Nurden, 2011). Platelets in metastatic cancer patients change the expression of VEGF, PF4 and TSP-1 with increase of platelet number and marks of persistent activation (Wiesner et al., 2010). VEGF is increased, while PF and TSP-1 are reduced, raising multiple hypotheses for the differentially released mediators, the platelets protein scavenging function, or the modulation of the megakaryopoiesis (megakaryocyte maturation and subsequent platelet formation from the common hemopoietic stem cell) (Geddis, 2010). It has been shown that platelet depletion, or the block of platelet surface receptors, decreases metastasis formation and tumor-platelets aggregates in animal models. This correlation between platelets and tumor metastasis is the shield hypothesis (Erpenbeck and Schon, 2010).

Embolization of tumor cells covered by platelets could be one of the ways for cancer spreading. Platelets may be an anchorage system by their subendothelial receptors and integrins (such as P-selectin, GPIb, αIIbβ3) to breakdown the neurovascular unit and defeat the ECM resistance through MMPs secretion, producing a shield against the immune system, releasing transforming growth factor beta (TGF-β) and inhibiting natural killer cell antitumor reactivity (Kim et al., 1998; Kopp et al., 2009). Tumor cells can promote thrombocytosis by interfering with the megakaryocytopoiesis or by releasing unknown inflammatory cytokines (Falanga et al., 2009). Cancer produces a well-known hypercoagulability state and thrombin can promote cancer growth and VEGF release supporting the vascular net growth in the expanding mass (Falanga et al., 2009). Platelets factors also play intriguing roles in promoting metastasis and favoring angiogenesis through VEGF release. Although platelets express anti-angiogenic factor, such as TSP-1, PF4 and endostatin (Zaslavsky et al., 2010), they are the main serum sources of these mediators (Browder et al., 2000; Folkman et al., 2001). In the recent years, extensive literature has been produced on the increase of these proteins in cancer, aiming to define whether it is specific of the early cancer stages or regulating tumor growth, however, the precise mechanism underlying metastasis and neuro-immune system crosslink remains to be explained (Nurden, 2011). From this perspective, the C-type lectin-like receptor 2 (CLEC-2)A plays a key role in mediating tumor interactions; it binds to podoplanin, physiologically expressed on kidney podocytes and lymphatic endothelial cells, protecting the tumor from the immune system aggression and inducing platelet aggregation and tumor metastasis progression (Suzuki-Inoue et al., 2007).

Gliomas are the most common primary CNS tumors (Louis et al., 2016). They are classified, by the World Health Organization (WHO), by their molecular markers, the biological activity and the histologic features, from grade I (non-malignant and slowly-growing associated with long-term survival) to grade IV (very aggressive malignant tumors, with fast-rate growing and short patient survival) (Popova et al., 2014; Louis et al., 2016). In adults, the glioblastoma multiforme (GBM) is the most frequent grade IV glioma characterized by highly infiltrative mass, with diffuse necrosis inside, highly infiltrative nature and whole brain diffusion, marked angiogenesis, apoptosis deregulation, and high genetic instability (Popova et al., 2014; Louis et al., 2016).

Thrombin levels into the lesion are very high, with a typical co-occurrence of thrombosis and hemorrhage that could in some extent favor the uncontrolled dissemination and growth of the malignant mass (Le Rhun and Perry, 2016; Mihara et al., 2016). The transduction-signaling pathway of thrombin, in these circumstances, is essentially by the canonical activation of PARs. Activation of PAR2, the lower affinity receptor, is possible, due to its high concentration found in brain tumor and following a vascular injury (Mihara et al., 2016). The putative PARs transactivation and dimerization could affect several cell functions by multiple transduction signals, still unknown but a promising gateway to design new therapeutic strategies (Mihara et al., 2016).

Tumor gene instability causing the development of cancer, whatever is the underlying mechanism or causative gene, leads to an altered cell proliferation/apoptosis ratio with the immortalization of a cellular clone. This process is, at final steps, regulated by mitogen activated protein kinases (MAPK), mainly extracellular signal–regulated kinase (ERK)1 and ERK2 (Nicole et al., 2005; Carmo et al., 2014). The intracellular transduction pathway seems to be cell-type related; however, the PI3K/PLC/PKC transduction can be considered as the key player (Wang and Reiser, 2003). PAR1, is even in these circumstances the crucial thrombin receptor and its expression, well-investigated in metastatic tumor, seems to be correlated to the tumor grading and invasiveness (Zhang et al., 2011), thus opening the research for future development of targeted chemotherapies (Kirwan et al., 2016). PARs can react with platelets beyond the tumor cells site. Platelets recognize also normal resident cells, not involved in the neoplastic process, and exert a neurotoxic effect on the healthy tissue, whereas tumor cells, expressing platelet receptors (mimicry phenomenon) can activate mitogenic pathways through the interaction between thrombin and PAR1-4 (Sotnikov et al., 2013; Wojtukiewicz et al., 2016). Surprisingly thrombin, at the site of the lesion, may mediate a protective role in a concentration independent manner. The protease can process the esophageal cancer related gene 4 (Ecrg4) having a chemotactic property for myeloid cells and can activate a host-versus-cancer pro-inflammatory reaction, slowing tumor growth (Lee et al., 2015), hastening the neuro-immune response. Ecrg4 that could in principle be the switch between thrombin adaptive/maladaptive changes and a key node in the neuro-immune activation is indeed downregulated in the high grade gliomas (Gotze et al., 2009).

Thrombin is involved in the persistent release of growth factors, as discussed for VEGF and tumor neo-angiogenesis (Hua et al., 2005). Fibrin, stabilized by FXIIIa is one of the proposed mechanism of the immunological masking (Walter et al., 2012). Kallicreins (Drucker et al., 2015), and specifically kallicreins 6-7-9, are also associated with poor survival in patients with GMB. How these serine proteases directly promote tumor cell survival need to be revealed, however, kallicrein 6 induces resistance to radiation and chemotherapy (temozolomide) in glioma cells by a PAR1 dependent mechanism (Drucker et al., 2013, 2015).

The TF, the principal initiator of coagulation, is also upregulated in gliomas and perilesional tissues (Walter et al., 2012; Le Rhun and Perry, 2016), hence being the activator of many thrombin-related toxic effects. Microglial activation has harmful effects on neurons and astrocytes (Zimmer et al., 2010) when exposed to high-level of thrombin and the reaction is characterized by intra and perilesional edema and altered leukocyte infiltration (Walter et al., 2012) or complement activation (Horstman et al., 2011). The complement system has been historically correlated to the cancer destruction mediated by the innate immune system, but it has been reported that a component of the system can promote the growth of malignant tumors (Markiewski and Lambris, 2009). The occurrence of C3 and C5b-9 complex depots in patients with GBM suggests the involvement of these proteins in the cancer process, even though serum level of complement system does not correlate with patient survival (Bouwens et al., 2015).

## Psychiatric Diseases

Mental health has many definitions. The WHO recognizes the “state of well-being” as the possibility to realize the person’s potential, resilience to the normal life stressors and, based on the social nature of our species, it emphasizes that for mental health recognition a person should make a working contribution to the community (Manwell et al., 2015). The absence of mental health, without detectable macroscopic organic lesion, is the domain of psychiatric diseases. The most treated and frequent disorders are unipolar depression, bipolar disorder, schizophrenia, and substance use disorders (addiction). Depression, expressed to a great extension among the population, is strictly related to blood coagulation disorders and complement activation (Thombs et al., 2006; Dantzer et al., 2008). The incidence of this disease grows from the 5% of the population up to 20% in subjects following ischemic heart diseases; moreover, cardiovascular diseases represent an independent risk factor for major depression (Forrester et al., 1992; Frasure-Smith et al., 1993; Thombs et al., 2006).

There are many variables to take into consideration when relating infarcts and depression. Obviously, scarce therapy compliance and negative habits (e.g., smoking, physical inactivity, dietary high calories intake) may influence both diseases; however, the role played by the underlying molecular pathways must be taken into account (Nemeroff and Musselman, 2000; Parissis et al., 2007). A 20-year study has shown that men which were hostile and prone to anger or depression had a complement system hyper-activation with the increase of C3, that could favor an ischemic heart disease. The study results were standardized for age, alcohol intake, and body mass index (BMI) (Boyle et al., 2007).

The relation between C3 increase and heart diseases has been confirmed in both myocardial infarctions and stable angina pectoris (Yan et al., 2016). In a mouse model of depression the levels of glial protein S100B and C3 has been found significantly lower in the amygdala, hippocampus, hypothalamus, prefrontal cortex, and striatum in control and after peripheral LPS administration (Strenn et al., 2015). The role of C3 in the development of CNS has been studied in C3 deficient mice; they showed reduced immature synapse elimination and development of aberrant neuronal networks (Stevens et al., 2007). In schizophrenic patients, in comparison to bipolar patients and healthy subjects, the C3 and C4 serum concentrations and their hemolytic properties were decreased (Spivak et al., 1993). A pathway proteomic profile of ischemic stroke survivors with mild depression showed that both lectin and the classical pathway of complement activation are downregulated and the phenomenon is associated with depressive symptoms (Nguyen et al., 2016). C3 is pivotal for the entire process; it is required for both lectin and classical activation of complement and for immune system regulation.

Platelets are the putative intriguing link interlacing mental, immune system, and coagulation-related disorders. These elements pivotal in the coagulation and complement cascade are the main reservoir of systemic serotonin. Indeed, their hyperactivity could explain both ischemic and depressive disorders (Nemeroff and Musselman, 2000; Musselman et al., 2002). Patients affected by major depressive disorder (MDD), matched with healthy subjects, showed increased platelet activation and expression of GPIIb-IIIa and P-selectin (Musselman et al., 1996). Platelets BDNF-containing granules were decreased in MDD, which could account for the limbic cortical thickness reduction (Duman and Monteggia, 2006; Liu et al., 2012). Treatment with the Citalopram, a selective serotonin reuptake inhibitor (SSRI), normalized BDNF level. Additionally, a single nucleotide polymorphism (SNP) of BDNF has been frequently associated with MDD, bipolar disorder (Sen et al., 2003), and coronary heart disease (Bozzini et al., 2009). It has been reported that the physiological response of platelets to thrombin is increased in manic, compared to depressed or schizophrenic patients, via a PKC modulated pathway (Wang et al., 1999). Mental health disorders are generally associated to a pro-coagulatory activity showing fibrinolytic residues in the general circulation (this is partly reversed by the therapy) (Schroeder et al., 2007; Geiser et al., 2011). The methylenetetrahydrofolate reductase (MTHFR) gene variant C677T, involved in the one-carbon metabolic pathway which is essential for DNA biosynthesis and the epigenetic process of DNA methylation, a cardiovascular and ischemic stroke independent risk factor, has been associated with schizophrenia, bipolar and unipolar depressive disorder (Peerbooms et al., 2011; El-Hadidy et al., 2014).

Psychoactive drugs interact with the CNS reward system and cause a substance abuse disorder (also known as addiction) interfering with the fine neurotransmitters balance (Goldstein and Volkow, 2002). Substance abuse disorder is a self-maintaining disorder with the dismal capacity of compulsive seeking repression, which overcome self-preserving mechanisms and the judgment of risk-taking (Hyman and Malenka, 2001). The social burden and the danger, deriving from the incapacity of measuring the negative outcome of actions, make addiction devastating, especially when it evolves in a chronic condition with the failure of the scarcely available treatments (O’Brien, 2011). Opiate abuse has been related to the deficiency of blood coagulation cascade with an increased fibrinolysis and higher levels of α2-macroglobulin (Ceriello et al., 1985). Morphine, methamphetamine, and nicotine increase the release of dopamine in the nucleus accumbens and activate post-synaptic dopamine receptors D1 (D1R). D1R activates cAMP/PKA pathway and extracellular enhancement of tPA activity in the nucleus accumbens that regulates dopamine release through PAR1 and is involved in the double mechanism of reward and dependence (Ito et al., 2007; Nagai et al., 2008). Ethanol addiction and withdrawal further enhance tPA activity. This mechanism is not plasmin-PAR1 mediated, but it produces the overexpression of NR2B subunit of the *N*-methyl-D-aspartate receptor (NMDAR) without intermediate proteolytic activity. Ethanol could inhibit the glutamate receptor whose expression is compensatively increased, until a rapid decrease of ethanol levels relieves the inhibitory tone on the overexpressed NMDAR population, leading to withdrawal symptoms and even seizures (Pawlak et al., 2005; Nagai et al., 2008).

## Perspective

Experimental and clinical reports unequivocally correlate the coagulation with the immune system and neuroinflammatory, degenerative, ischemic, proliferative, and functional disorders of the CNS. Platelet-derived, complement and clotting factors, CNS resident cells and protease intracellular signal transduction can modify the neurovascular unit integrity and recruit leukocytes at the site of the damage (Figure 2). Circulating coagulation proteins, complement and immune cells interact with several messengers and with each other to provide an active continuous scavenging activity, promptly correcting the structural or functional failures that constantly jeopardize the CNS homeostasis.

Central nervous system elements are partially protected by the immune privilege guaranteed by the BBB, through a constantly and actively filtering operated by endothelium, astrocytes, pericytes, and ECM. However, they express all the potential ligand and receptors to react actively to external damage. The chronicity of this reaction, mediated by both endogenous as exogenous elements can partly account for the switch between a remedial adaptive plasticity to the damage and a maladaptive rewiring of the CNS with disruption of synaptic stability and aberrant formation of newly deposed ECM. In the neuro-immune hemostasis framework concurring to CNS pathology, the coagulation and the immune system are cooperative in pathophysiological conditions, specifically after the BBB failure.

**FIGURE 3:**
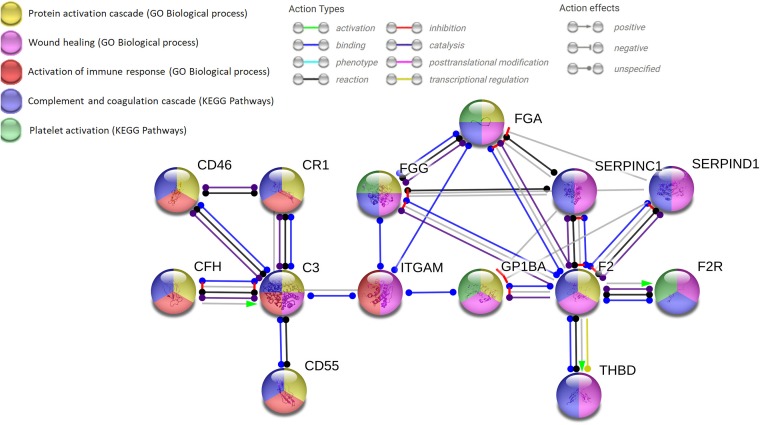
Network analysis using the string.org platform for protein–protein interactions (PPIs) which integrates Gene Ontology (GO) and Kyoto Encyclopedia of Genes and Genomes (KEGG) database. The databases query demonstrates the solid neuro-immune hemostatic network with the pivotal hubs strictly interconnected and the expandable hub-spoke associations. Four input proteins (F2, C3, ITGAM, GP1BA) with 14 nodes and 23 edges (minimum required interaction score: highest confidence 0.900; PP1 enrichment *p*-value of 0.00103). C3, complement component 3; CD, cluster of differentiation; CFH, complement factor H; CR1, complement component (3b/4b) receptor 1; FG, fibrinogen: alpha (FGA) or gamma (FGG) chain; F2, thrombin; F2R, protease-activated receptor-1 (PAR-1); ITGAM, integrin αM (C3 receptor 3 subunit); THBD, thrombomodulin; SERPINC1, serpin peptidase inhibitor, clade C member 1 (antithrombin III); SERPIND1, serpin peptidase inhibitor, clade D member 1 (heparan cofactor).

The analysis of literature data on cellular-protein-interactions involved in neuro-immune hemostasis allows designing a proposal for the main protein-hubs of this network formed by three fundamental nodes and one internode (Figure 3). The first node of the network is thrombin with activation of PAR-1 in a dose-dependent manner; the second node is C3, linking coagulation, synaptic plasticity and immune system; the third node is GPIb, essential for activated platelet anchorage, coagulation processes, thrombin activation and C3 activation. The internode is represented by αMβ2 integrin (part of the CR3) connecting the second and the third node of the network.

To better understand how to favor the maintenance of the remedial adaptive plasticity, which would have a clear therapeutic interest for several pathological conditions, it would be useful to construct predictive molecular models, like the proposed network. The perspective is to be able to enlighten the regulating logic of the complex molecular network of Figure 3, which belongs to different cellular domains (neurons, astrocytes, BBB, and blood cells).

Several types of mathematical models have been described in the literature to analyze complex patho/physiological functions. The more immediate tool is offered by network analysis (Loscalzo et al., 2017), which builds on protein–protein interaction map (PPI), performed at genome-wide level. For several diseases it has been reported where the nodes of the network under examination (in our case those of Figure 3) cluster on the general PPI map. In this way, it would be possible to ascertain which basic cellular functions interact with the cluster under investigation. It would be of interest, for instance, to know whether one of the nodes in Figure 3 interacts with mitochondrial metabolism, autophagy or apoptosis.

Other possibilities are offered by dynamic systems biology models (Abudukelimu et al., 2018; Barberis et al., 2018), whose computational analysis has been able to shed some light on the regulatory logic governing either inflammation response or T cell differentiation. It is expected that the interactions of computational models and new experiments, suggested by the model predictions, will be able to elucidate the fine structure of molecular events generated by blood coagulation and neuro-immune response in several CNS diseases, thereby opening the way to more effective treatments.

## Author Contributions

CDL wrote the first draft of the manuscript, managed the references, and prepared the figures. AC wrote part of the manuscript, made constructive changes, and revised the final text of the manuscript. LA gave conceptual contribution to the manuscript. MP conceived, designed, wrote, and revised the manuscript. All authors read and approved the final version of the manuscript.

## Conflict of Interest Statement

The authors declare that the research was conducted in the absence of any commercial or financial relationships that could be construed as a potential conflict of interest.

## Footnotes

1 https://clinicaltrials.gov/ct2/show/NCT01892345

2 https://clinicaltrials.gov/ct2/show/NCT02002390

3 https://clinicaltrials.gov/ct2/show/NCT02956200

## References

[B1] Abudukelimu A. Barberis M. Redegeld F. A. Sahin N. Westerhoff H. V. (2018). Predictable irreversible switching between acute and chronic inflammation. *Front. Immunol.* 7:1596. 10.3389/fimmu.2018.01596 30131800 PMC6090016

[B2] Adams R. A. Bauer J. Flick M. J. Sikorski S. L. Nuriel T. Lassmann H. (2007). The fibrin-derived gamma377-395 peptide inhibits microglia activation and suppresses relapsing paralysis in central nervous system autoimmune disease. *J. Exp. Med.* 204 571–582. 10.1084/jem.20061931 17339406 PMC2137908

[B3] Akassoglou K. Adams R. A. Bauer J. Mercado P. Tseveleki V. Lassmann H. (2004). Fibrin depletion decreases inflammation and delays the onset of demyelination in a tumor necrosis factor transgenic mouse model for multiple sclerosis. *Proc. Natl. Acad. Sci. U.S.A.* 101 6698–6703. 10.1073/pnas.0303859101 15096619 PMC404108

[B4] Alabanza L. M. Esmon N. L. Esmon C. T. Bynoe M. S. (2013). Inhibition of endogenous activated protein C attenuates experimental autoimmune encephalomyelitis by inducing myeloid-derived suppressor cells. *J. Immunol.* 191 3764–3777. 10.4049/jimmunol.1202556 23997223 PMC3800123

[B5] Ambrosini E. Columba-Cabezas S. Serafini B. Muscella A. Aloisi F. (2003). Astrocytes are the major intracerebral source of macrophage inflammatory protein-3α/CCL20 in relapsing experimental autoimmune encephalomyelitis and in vitro. *Glia* 41 290–300. 10.1002/glia.10193 12528183

[B6] Arachiche A. Mumaw M. M. de la Fuente M. Nieman M. T. (2013). Protease-activated Receptor 1 (PAR1) and PAR4 Heterodimers Are Required for PAR1-enhanced Cleavage of PAR4 by α-Thrombin. *J. Biol. Chem.* 288 32553–32562. 10.1074/jbc.M113.472373 24097976 PMC3820888

[B7] Bahou W. F. (2013). Genetic dissection of platelet function in health and disease using systems biology. *Hematol. Oncol. Clin. North. Am.* 27 443–463. 10.1016/j.hoc.2013.03.002 23714307 PMC3767180

[B8] Baloyannis S. J. (2015). Brain capillaries in Alzheimer’s disease. *Hell. J. Nucl. Med.* 18(Suppl. 1), 152 10.15406/jnsk.2015.02.00069 26665235

[B9] Barberis M. Helikar T. Verbruggen P. (2018). Simulation of stimulation: cytokine dosage and cell cycle crosstalk driving timing-dependent T cell differentiation. *Front. Physiol.* 2:879. 10.3389/fphys.2018.00879 30116196 PMC6083814

[B10] Barbier M. Faille D. Loriod B. Textoris J. Camus C. Puthier D. (2011). Platelets alter gene expression profile in human brain endothelial cells in an in vitro model of cerebral malaria. *PLoS One* 6:e19651. 10.1371/journal.pone.0019651 21603600 PMC3095604

[B11] Bartanusz V. Jezova D. Alajajian B. Digicaylioglu M. (2011). The blood-spinal cord barrier: morphology and clinical implications. *Ann. Neurol.* 70 194–206. 10.1002/ana.22421 21674586

[B12] Becker D. Ikenberg B. Schiener S. Maggio N. Vlachos A. (2014). NMDA-receptor inhibition restores Protease-Activated Receptor 1 (PAR1) mediated alterations in homeostatic synaptic plasticity of denervated mouse dentate granule cells. *Neuropharmacology* 86 212–218. 10.1016/j.neuropharm.2014.07.013 25086265

[B13] Bedoui Y. Neal J. W. Gasque P. (2018). The Neuro-Immune-Regulators (NIREGs) Promote Tissue Resilience; a Vital Component of the Host’s Defense Strategy against Neuroinflammation. *J. Neuroimmune Pharmacol.* 13 309–329. 10.1007/s11481-018-9793-6 29909495

[B14] Beggs S. Salter M. W. (2016). SnapShot: microglia in disease. *Cell* 165 1294–1294.e1. 10.1016/j.cell.2016.05.036 27203115

[B15] Belbasis L. Bellou V. Evangelou E. Ioannidis J. P. Tzoulaki I. (2015). Environmental risk factors and multiple sclerosis: an umbrella review of systematic reviews and meta-analyses. *Lancet Neurol.* 14 263–273. 10.1016/s1474-4422(14)70267-4 25662901

[B16] Ben Khalifa N. Tyteca D. Marinangeli C. Depuydt M. Collet J. F. Courtoy P. J. (2012). Structural features of the KPI domain control APP dimerization, trafficking, and processing. *FASEB J.* 26 855–867. 10.1096/fj.11-190207 22085646

[B17] Ben Shimon M. Lenz M. Ikenberg B. Becker D. Shavit Stein E. Chapman J. (2015). Thrombin regulation of synaptic transmission and plasticity: implications for health and disease. *Front. Cell Neurosci.* 9:151. 10.3389/fncel.2015.00151 25954157 PMC4404867

[B18] Bouwens T. A. Trouw L. A. Veerhuis R. Dirven C. M. Lamfers M. L. Al-Khawaja H. (2015). Complement activation in Glioblastoma multiforme pathophysiology: evidence from serum levels and presence of complement activation products in tumor tissue. *J. Neuroimmunol.* 278 271–276. 10.1016/j.jneuroim.2014.11.016 25468776

[B19] Boyle S. H. Jackson W. G. Suarez E. C. (2007). Hostility, anger, and depression predict increases in C3 over a 10-year period. *Brain Behav. Immun.* 21 816–823. 10.1016/j.bbi.2007.01.008 17321106 PMC1995457

[B20] Bozzini S. Gambelli P. Boiocchi C. Schirinzi S. Falcone R. Buzzi P. (2009). Coronary artery disease and depression: possible role of brain-derived neurotrophic factor and serotonin transporter gene polymorphisms. *Int. J. Mol. Med.* 24 813–818. 10.3892/ijmm_00000297 19885623

[B21] Browder T. Folkman J. Pirie-Shepherd S. (2000). The hemostatic system as a regulator of angiogenesis. *J. Biol. Chem.* 275 1521–1524. 10.1074/jbc.275.3.1521 10636838

[B22] Brown G. T. Narayanan P. Li W. Silverstein R. L. McIntyre T. M. (2013). Lipopolysaccharide stimulates platelets through an IL-1beta autocrine loop. *J. Immunol.* 191 5196–5203. 10.4049/jimmunol.1300354 24081990 PMC3818355

[B23] Bruno M. A. Cuello A. C. (2006). Activity-dependent release of precursor nerve growth factor, conversion to mature nerve growth factor, and its degradation by a protease cascade. *Proc. Natl. Acad. Sci. U.S.A.* 103 6735–6740. 10.1073/pnas.0510645103 16618925 PMC1458950

[B24] Buisson A. Nicole O. Docagne F. Sartelet H. Mackenzie E. T. Vivien D. (1998). Up-regulation of a serine protease inhibitor in astrocytes mediates the neuroprotective activity of transforming growth factor beta1. *FASEB J.* 12 1683–1691. 10.1096/fasebj.12.15.1683 9837858

[B25] Burneo J. G. Fang J. Saposnik G. (2010). Impact of seizures on morbidity and mortality after stroke: a Canadian multi-centre cohort study. *Eur. J. Neurol.* 17 52–58. 10.1111/j.1468-1331.2009.02739.x 19686350

[B26] Bushi D. Ben Shimon M. Shavit Stein E. Chapman J. Maggio N. Tanne D. (2015). Increased thrombin activity following reperfusion after ischemic stroke alters synaptic transmission in the hippocampus. *J. Neurochem.* 135 1140–1148. 10.1111/jnc.13372 26390857

[B27] Carmo A. A. Costa B. R. Vago J. P. de Oliveira L. C. Tavares L. P. Nogueira C. R. (2014). Plasmin induces in vivo monocyte recruitment through protease-activated receptor-1-, MEK/ERK-, and CCR2-mediated signaling. *J. Immunol.* 193 3654–3663. 10.4049/jimmunol.1400334 25165151

[B28] Carvalho-Tavares J. Hickey M. J. Hutchison J. Michaud J. Sutcliffe I. T. Kubes P. (2000). A role for platelets and endothelial selectins in tumor necrosis factor-alpha-induced leukocyte recruitment in the brain microvasculature. *Circ. Res.* 87 1141–1148. 10.1161/01.RES.87.12.1141 11110771

[B29] Ceriello A. Dello Russo P. Curcio F. Passariello N. Giugliano D. (1985). Increased alpha-2-macroglobulin in opiate addicts: further evidence of an alteration in the coagulation system due to opiate addiction. *Acta Haematol.* 73:117. 10.1159/000206295 2409723

[B30] Chen N.-F. Sung C.-S. Wen Z.-H. Chen C.-H. Feng C.-W. Hung H.-C. (2018). Therapeutic effect of platelet-rich plasma in rat spinal cord injuries. *Front. Neurosci.* 12:252. 10.3389/fnins.2018.00252 29740270 PMC5924817

[B31] Cheng T. Liu D. Griffin J. H. Fernandez J. A. Castellino F. Rosen E. D. (2003). Activated protein C blocks p53-mediated apoptosis in ischemic human brain endothelium and is neuroprotective. *Nat. Med.* 9 338–342. 10.1038/nm826 12563316

[B32] Cirillo G. Colangelo A. M. De Luca C. Savarese L. Barillari M. R. Alberghina L. (2016). Modulation of matrix metalloproteinases activity in the ventral horn of the spinal cord re-stores neuroglial synaptic homeostasis and neurotrophic support following peripheral nerve injury. *PLoS One* 11:e0152750. 10.1371/journal.pone.0152750 27028103 PMC4814041

[B33] Cofiell R. Kukreja A. Bedard K. Yan Y. Mickle A. P. Ogawa M. (2015). Eculizumab reduces complement activation, inflammation, endothelial damage, thrombosis, and renal injury markers in aHUS. *Blood* 125 3253–3262. 10.1182/blood-2014-09-600411 25833956 PMC4449039

[B34] Colangelo A. M. Cirillo G. Lavitrano M. L. Alberghina L. Papa M. (2012). Targeting reactive astrogliosis by novel biotechnological strategies. *Biotechnol. Adv.* 30 261–271. 10.1016/j.biotechadv.2011.06.016 21763415

[B35] Colangelo A. M. Mallei A. Johnson P. F. Mocchetti I. (2004). Synergistic effect of dexamethasone and beta-adrenergic receptor agonists on the nerve growth factor gene transcription. *Brain Res. Mol. Brain Res.* 124 97–104. 10.1016/j.molbraines.2004.01.011 15135217

[B36] Cosgrove L. J. d’Apice A. J. Haddad A. Pedersen J. McKenzie I. F. (1987). CR3 receptor on platelets and its role in the prostaglandin metabolic pathway. *Immunol. Cell Biol.* 65( Pt 6), 453–460. 10.1038/icb.1987.54 2966106

[B37] Coughlin S. R. (2000). Thrombin signalling and protease-activated receptors. *Nature* 407 258–264. 10.1038/35025229 11001069

[B38] Coughlin S. R. (2005). Protease-activated receptors in hemostasis, thrombosis and vascular biology. *J. Thromb. Haemost.* 3 1800–1814. 10.1111/j.1538-7836.2005.01377.x 16102047

[B39] Cuello A. C. Bruno M. A. Allard S. Leon W. Iulita M. F. (2009). Cholinergic involvement in Alzheimer’s disease. A link with NGF maturation and degradation. *J. Mol. Neurosci.* 40 230–235. 10.1007/s12031-009-9238-z 19680822

[B40] Dahdaleh M. Alroughani R. Aljumah M. AlTahan A. Alsharoqi I. Bohlega S. A. (2017). Intervening to reduce the risk of future disability from multiple sclerosis: are we there yet? *Int. J. Neurosci.* 127 944–951. 10.1080/00207454.2016.1277424 28029270

[B41] Dantzer R. O’Connor J. C. Freund G. G. Johnson R. W. Kelley K. W. (2008). From inflammation to sickness and depression: when the immune system subjugates the brain. *Nat. Rev. Neurosci.* 9 46–56. 10.1038/nrn2297 18073775 PMC2919277

[B42] de Jager M. Drukarch B. Hofstee M. Breve J. Jongenelen C. A. Bol J. G. (2015). Tissue transglutaminase-catalysed cross-linking induces Apolipoprotein E multimers inhibiting Apolipoprotein E’s protective effects towards amyloid-beta-induced toxicity. *J. Neurochem.* 134 1116–1128. 10.1111/jnc.13203 26088696

[B43] De Luca C. Papa M. (2016). Looking inside the matrix: perineuronal nets in plasticity, maladaptive plasticity and neurological disorders. *Neurochem. Res.* 41 1507–1515. 10.1007/s11064-016-1876-2 26935742

[B44] De Luca C. Papa M. (2017). Matrix metalloproteinases, neural extracellular matrix, and central nervous system pathology. *Prog. Mol. Biol. Transl. Sci.* 148 167–202. 10.1016/bs.pmbts.2017.04.002 28662822

[B45] De Luca C. Virtuoso A. Maggio N. Papa M. (2017). Neuro-coagulopathy: blood coagulation factors in central nervous system diseases. *Int. J. Mol. Sci.* 18:E2128. 10.3390/ijms18102128 29023416 PMC5666810

[B46] de Robles P. Fiest K. M. Frolkis A. D. Pringsheim T. Atta C. St Germaine-Smith C. (2015). The worldwide incidence and prevalence of primary brain tumors: a systematic review and meta-analysis. *Neuro Oncol.* 17 776–783. 10.1093/neuonc/nou283 25313193 PMC4483114

[B47] Dhuriya Y. K. Sharma D. (2018). Necroptosis: a regulated inflammatory mode of cell death. *J. Neuroinflammation* 15:199. 10.1186/s12974-018-1235-0 29980212 PMC6035417

[B48] Dityatev A. Rusakov D. A. (2011). Molecular signals of plasticity at the tetrapartite synapse. *Curr. Opin. Neurobiol.* 21 353–359. 10.1016/j.conb.2010.12.006 21277196 PMC3368316

[B49] Drucker K. L. Gianinni C. Decker P. A. Diamandis E. P. Scarisbrick I. A. (2015). Prognostic significance of multiple kallikreins in high-grade astrocytoma. *BMC Cancer* 15:565. 10.1186/s12885-015-1566-5 26231762 PMC4521496

[B50] Drucker K. L. Paulsen A. R. Giannini C. Decker P. A. Blaber S. I. Blaber M. (2013). Clinical significance and novel mechanism of action of kallikrein 6 in glioblastoma. *Neuro Oncol.* 15 305–318. 10.1093/neuonc/nos313 23307575 PMC3578488

[B51] Dubois B. Feldman H. H. Jacova C. Hampel H. Molinuevo J. L. Blennow K. (2014). Advancing research diagnostic criteria for Alzheimer’s disease: the IWG-2 criteria. *Lancet Neurol.* 13 614–629. 10.1016/s1474-4422(14)70090-0 24849862

[B52] Duman R. S. Monteggia L. M. (2006). A neurotrophic model for stress-related mood disorders. *Biol. Psychiatry* 59 1116–1127. 10.1016/j.biopsych.2006.02.013 16631126

[B53] Ehrlich P. (1900). Croonian lecture.—On immunity with special reference to cell life. *Proc. R. Soc. Lond.* 66 424–448. 10.1098/rspl.1899.0121

[B54] El-Hadidy M. A. Abdeen H. M. Abd El-Aziz S. M. Al-Harrass M. (2014). MTHFR gene polymorphism and age of onset of schizophrenia and bipolar disorder. *BioMed Res. Int.* 2014:318483. 10.1155/2014/318483 25101272 PMC4101969

[B55] Erpenbeck L. Schon M. P. (2010). Deadly allies: the fatal interplay between platelets and metastasizing cancer cells. *Blood* 115 3427–3436. 10.1182/blood-2009-10-247296 20194899 PMC2867258

[B56] Evin G. Zhu A. Holsinger R. M. Masters C. L. Li Q. X. (2003). Proteolytic processing of the Alzheimer’s disease amyloid precursor protein in brain and platelets. *J. Neurosci. Res.* 74 386–392. 10.1002/jnr.10745 14598315

[B57] Falanga A. Panova-Noeva M. Russo L. (2009). Procoagulant mechanisms in tumour cells. *Best Pract. Res. Clin. Haematol.* 22 49–60. 10.1016/j.beha.2008.12.009 19285272

[B58] Feinstein A. Freeman J. Lo A. C. (2015). Treatment of progressive multiple sclerosis: what works, what does not, and what is needed. *Lancet Neurol.* 14 194–207. 10.1016/s1474-4422(14)70231-5 25772898

[B59] Festoff B. W. Sajja R. K. van Dreden P. Cucullo L. (2016). HMGB1 and thrombin mediate the blood-brain barrier dysfunction acting as biomarkers of neuroinflammation and progression to neurodegeneration in Alzheimer’s disease. *J. Neuroinflammation* 13 194–194. 10.1186/s12974-016-0670-z 27553758 PMC4995775

[B60] Folkman J. Browder T. Palmblad J. (2001). Angiogenesis research: guidelines for translation to clinical application. *Thromb. Haemost.* 86 23–33. 10.1055/s-0037-1616197 11487011

[B61] Forrester A. W. Lipsey J. R. Teitelbaum M. L. DePaulo J. R. Andrzejewski P. L. (1992). Depression following myocardial infarction. *Int. J. Psychiatry Med.* 22 33–46. 10.2190/cj9d-32c2-8cm7-ft3d 1577547

[B62] Frasure-Smith N. Lesperance F. Talajic M. (1993). Depression following myocardial infarction. Impact on 6-month survival. *JAMA* 270 1819–1825. 10.1001/jama.1993.03510150053029 8411525

[B63] Gallego Perez-Larraya J. Hildebrand J. (2014). Brain metastases. *Handb. Clin. Neurol.* 121 1143–1157. 10.1016/b978-0-7020-4088-7.00077-8 24365409

[B64] Garcia P. S. Ciavatta V. T. Fidler J. A. Woodbury A. Levy J. H. Tyor W. R. (2015). Concentration-dependent dual role of thrombin in protection of cultured rat cortical neurons. *Neurochem. Res.* 40 2220–2229. 10.1007/s11064-015-1711-1 26342829 PMC4644093

[B65] García-Berrocoso T. Giralt D. Llombart V. Bustamante A. Penalba A. Flores A. (2014). Chemokines after human ischemic stroke: from neurovascular unit to blood using protein arrays. *Transl. Proteom.* 3 1–9. 10.1016/j.trprot.2014.03.001

[B66] GBD 2015 Mortality and Causes of Death Collaborators (2016). Global, regional, and national life expectancy, all-cause mortality, and cause-specific mortality for 249 causes of death, 1980-2015: a systematic analysis for the Global Burden of Disease Study 2015. *Lancet* 388 1459–1544. 10.1016/s0140-6736(16)31012-1 27733281 PMC5388903

[B67] Geddis A. E. (2010). Megakaryopoiesis. *Semin. Hematol.* 47 212–219. 10.1053/j.seminhematol.2010.03.001 20620431 PMC2904992

[B68] Geiser F. Conrad R. Imbierowicz K. Meier C. Liedtke R. Klingmuller D. (2011). Coagulation activation and fibrinolysis impairment are reduced in patients with anxiety and depression when medicated with serotonergic antidepressants. *Psychiatry Clin. Neurosci.* 65 518–525. 10.1111/j.1440-1819.2011.02241.x 21851461

[B69] Giangrande P. (2003). Six characters in search of an author: the history of the nomenclature of coagulation factors. *Br. J. Haematol.* 121 703–712. 10.1046/j.1365-2141.2003.04333.x 12780784

[B70] Goldstein R. Z. Volkow N. D. (2002). Drug addiction and its underlying neurobiological basis: neuroimaging evidence for the involvement of the frontal cortex. *Am. J. Psychiatry* 159 1642–1652. 10.1176/appi.ajp.159.10.1642 12359667 PMC1201373

[B71] Gotze S. Feldhaus V. Traska T. Wolter M. Reifenberger G. Tannapfel A. (2009). ECRG4 is a candidate tumor suppressor gene frequently hypermethylated in colorectal carcinoma and glioma. *BMC Cancer* 9:447. 10.1186/1471-2407-9-447 20017917 PMC2804712

[B72] Guth J. C. Gerard E. E. Nemeth A. J. Liotta E. M. Prabhakaran S. Naidech A. M. (2014). Subarachnoid extension of hemorrhage is associated with early seizures in primary intracerebral hemorrhage. *J. Stroke Cerebrovasc. Dis.* 23 2809–2813. 10.1016/j.jstrokecerebrovasdis.2014.07.023 25194742

[B73] Hamad O. A. Nilsson P. H. Wouters D. Lambris J. D. Ekdahl K. N. Nilsson B. (2010). Complement component C3 binds to activated normal platelets without preceding proteolytic activation and promotes binding to complement receptor 1. *J. Immunol.* 184 2686–2692. 10.4049/jimmunol.0902810 20139276 PMC2953618

[B74] Hamill C. E. Mannaioni G. Lyuboslavsky P. Sastre A. A. Traynelis S. F. (2009). Protease-activated receptor 1-dependent neuronal damage involves NMDA receptor function. *Exp. Neurol.* 217 136–146. 10.1016/j.expneurol.2009.01.023 19416668 PMC2679858

[B75] Hartz A. M. Bauer B. Soldner E. L. Wolf A. Boy S. Backhaus R. (2012). Amyloid-beta contributes to blood-brain barrier leakage in transgenic human amyloid precursor protein mice and in humans with cerebral amyloid angiopathy. *Stroke* 43 514–523. 10.1161/strokeaha.111.627562 22116809 PMC5761312

[B76] Horstman L. L. Jy W. Ahn Y. S. Maghzi A. H. Etemadifar M. Alexander J. S. (2011). Complement in neurobiology. *Front. Biosci.* 16 2921–2960. 10.2741/3890 21622213

[B77] Hua Y. Tang L. Keep R. F. Schallert T. Fewel M. E. Muraszko K. M. (2005). The role of thrombin in gliomas. *J. Thromb. Haemost.* 3 1917–1923. 10.1111/j.1538-7836.2005.01446.x 15975137

[B78] Hultman K. Cortes-Canteli M. Bounoutas A. Richards A. T. Strickland S. Norris E. H. (2014). Plasmin deficiency leads to fibrin accumulation and a compromised inflammatory response in the mouse brain. *J. Thromb. Haemost.* 12 701–712. 10.1111/jth.12553 24612416 PMC4120644

[B79] Hyman S. E. Malenka R. C. (2001). Addiction and the brain: the neurobiology of compulsion and its persistence. *Nat. Rev. Neurosci.* 2 695–703. 10.1038/35094560 11584307

[B80] Italiano J. E. Jr. Richardson J. L. Patel-Hett S. Battinelli E. Zaslavsky A. Short S. (2008). Angiogenesis is regulated by a novel mechanism: pro- and antiangiogenic proteins are organized into separate platelet alpha granules and differentially released. *Blood* 111 1227–1233. 10.1182/blood-2007-09-113837 17962514 PMC2214735

[B81] Ito M. Nagai T. Mizoguchi H. Fukakusa A. Nakanishi Y. Kamei H. (2007). Possible involvement of protease-activated receptor-1 in the regulation of morphine-induced dopamine release and hyperlocomotion by the tissue plasminogen activator-plasmin system. *J. Neurochem.* 101 1392–1399. 10.1111/j.1471-4159.2006.04423.x 17286591

[B82] Joshi N. Kopec A. K. Ray J. L. Cline-Fedewa H. Nawabi A. Schmitt T. (2016). Fibrin deposition following bile duct injury limits fibrosis through an alphaMbeta2-dependent mechanism. *Blood* 127 2751–2762. 10.1182/blood-2015-09-670703 26921287 PMC4891955

[B83] Jun G. Naj A. C. Beecham G. W. Wang L. S. Buros J. Gallins P. J. (2010). Meta-analysis confirms CR1, CLU, and PICALM as alzheimer disease risk loci and reveals interactions with APOE genotypes. *Arch. Neurol.* 67 1473–1484. 10.1001/archneurol.2010.201 20697030 PMC3048805

[B84] Junge C. E. Lee C. J. Hubbard K. B. Zhang Z. Olson J. J. Hepler J. R. (2004). Protease-activated receptor-1 in human brain: localization and functional expression in astrocytes. *Exp. Neurol.* 188 94–103. 10.1016/j.expneurol.2004.02.018 15191806

[B85] Kavanagh D. Goodship T. H. Richards A. (2013). Atypical hemolytic uremic syndrome. *Semin. Nephrol.* 33 508–530. 10.1016/j.semnephrol.2013.08.003 24161037 PMC3863953

[B86] Kim Y. J. Borsig L. Varki N. M. Varki A. (1998). P-selectin deficiency attenuates tumor growth and metastasis. *Proc. Natl. Acad. Sci. U.S.A.* 95 9325–9330. 10.1073/pnas.95.16.9325 9689079 PMC21337

[B87] Kirwan C. C. Bundred N. J. Castle J. Clarke R. Dive C. Morris J. (2016). PO-36 - Thrombin Inhibition Preoperatively (TIP) in early breast cancer, the first clinical trial of NOACs as an anti-cancer agent: trial methodology. *Thromb. Res.* 140(Suppl. 1), S189–S190. 10.1016/s0049-3848(16)30169-4 27161723

[B88] Kopp H. G. Placke T. Salih H. R. (2009). Platelet-derived transforming growth factor-beta down-regulates NKG2D thereby inhibiting natural killer cell antitumor reactivity. *Cancer Res.* 69 7775–7783. 10.1158/0008-5472.can-09-2123 19738039

[B89] Kurabayashi H. Kubota K. Hishinuma A. Majima M. (2010). Platelet activation is caused not by aging but by atherosclerosis. *Arch. Gerontol. Geriatr.* 51 205–208. 10.1016/j.archger.2009.10.009 19932514

[B90] Kurabayashi H. Tamura J. Naruse T. Kubota K. (2000). Possible existence of platelet activation before the onset of cerebral infarction. *Atherosclerosis* 153 203–207. 10.1016/S0021-9150(00)00399-3 11058716

[B91] Lampron A. Elali A. Rivest S. (2013). Innate immunity in the CNS: redefining the relationship between the CNS and Its environment. *Neuron* 78 214–232. 10.1016/j.neuron.2013.04.005 23622060

[B92] Langer H. F. Choi E. Y. Zhou H. Schleicher R. Chung K. J. Tang Z. (2012). Platelets contribute to the pathogenesis of experimental autoimmune encephalomyelitis. *Circ. Res.* 110 1202–1210. 10.1161/circresaha.111.256370 22456181 PMC3382058

[B93] Le Rhun E. Perry J. R. (2016). Vascular complications in glioma patients. *Handb. Clin. Neurol.* 134 251–266. 10.1016/b978-0-12-802997-8.00015-3 26948359

[B94] Lee J. Dang X. Borboa A. Coimbra R. Baird A. Eliceiri B. P. (2015). Thrombin-processed Ecrg4 recruits myeloid cells and induces antitumorigenic inflammation. *Neuro Oncol.* 17 685–696. 10.1093/neuonc/nou302 25378632 PMC4482854

[B95] Lee P. R. Johnson T. P. Gnanapavan S. Giovannoni G. Wang T. Steiner J. P. (2017). Protease-activated receptor-1 activation by granzyme B causes neurotoxicity that is augmented by interleukin-1beta. *J. Neuroinflammation* 14:131. 10.1186/s12974-017-0901-y 28655310 PMC5488439

[B96] Lee R. Kermani P. Teng K. K. Hempstead B. L. (2001). Regulation of cell survival by secreted proneurotrophins. *Science* 30 1945–1948. 10.1126/science.1065057 11729324

[B97] Lenz M. Vlachos A. Maggio N. (2015). Ischemic long-term-potentiation (iLTP): perspectives to set the threshold of neural plasticity toward therapy. *Neural. Regen. Res.* 10 1537–1539. 10.4103/1673-5374.165215 26692832 PMC4660728

[B98] Lin C. C. Lee I. T. Chi P. L. Hsieh H. L. Cheng S. E. Hsiao L. D. (2014). C-Src/Jak2/PDGFR/PKCdelta-dependent MMP-9 induction is required for thrombin-stimulated rat brain astrocytes migration. *Mol. Neurobiol.* 49 658–672. 10.1007/s12035-013-8547-y 24018979

[B99] Lin C. C. Lee I. T. Wu W. B. Liu C. J. Hsieh H. L. Hsiao L. D. (2013). Thrombin mediates migration of rat brain astrocytes via PLC, Ca(2)(+), CaMKII, PKCalpha, and AP-1-dependent matrix metalloproteinase-9 expression. *Mol. Neurobiol.* 48 616–630. 10.1007/s12035-013-8450-6 23585120

[B100] Lin H. Trejo J. (2013). Transactivation of the PAR1-PAR2 heterodimer by thrombin elicits beta-arrestin-mediated endosomal signaling. *J. Biol. Chem.* 288 11203–11215. 10.1074/jbc.M112.439950 23476015 PMC3630854

[B101] Lindsberg P. J. Ohman J. Lehto T. Karjalainen-Lindsberg M. L. Paetau A. Wuorimaa T. (1996). Complement activation in the central nervous system following blood-brain barrier damage in man. *Ann. Neurol.* 40 587–596. 10.1002/ana.410400408 8871578

[B102] Liu C. Y. Jiang X. X. Zhu Y. H. Wei D. N. (2012). Metabotropic glutamate receptor 5 antagonist 2-methyl-6-(phenylethynyl)pyridine produces antidepressant effects in rats: role of brain-derived neurotrophic factor. *Neuroscience* 223 219–224. 10.1016/j.neuroscience.2012.08.010 22890078

[B103] Liu J. Zhang C. Tao W. Liu M. (2013). Systematic review and meta-analysis of the efficacy of sphingosine-1-phosphate (S1P) receptor agonist FTY720 (fingolimod) in animal models of stroke. *Int. J. Neurosci.* 123 163–169. 10.3109/00207454.2012.749255 23167788

[B104] Lock C. Hermans G. Pedotti R. Brendolan A. Schadt E. Garren H. (2002). Gene-microarray analysis of multiple sclerosis lesions yields new targets validated in autoimmune encephalomyelitis. *Nat. Med.* 8 500–508. 10.1038/nm0502-500 11984595

[B105] Lodygin D. Odoardi F. Schlager C. Korner H. Kitz A. Nosov M. (2013). A combination of fluorescent NFAT and H2B sensors uncovers dynamics of T cell activation in real time during CNS autoimmunity. *Nat. Med.* 19 784–790. 10.1038/nm.3182 23624600

[B106] Loscalzo J. Barabasi A. Silverman E. K. (2017). *Network Medicine: Complex Systems in Human Disease and Therapeutics*. Cambridge, MA: Harvard University Press.

[B107] Louis D. N. Perry A. Reifenberger G. von Deimling A. Figarella-Branger D. Cavenee W. K. (2016). The 2016 World Health Organization Classification of Tumors of the Central Nervous System: a summary. *Acta Neuropathol.* 131 803–820. 10.1007/s00401-016-1545-1 27157931

[B108] Lublin F. D. Reingold S. C. (1996). Defining the clinical course of multiple sclerosis: results of an international survey. National Multiple Sclerosis Society (USA) Advisory Committee on Clinical Trials of New Agents in Multiple Sclerosis. *Neurology* 46 907–911. 10.1212/WNL.46.4.907 8780061

[B109] Luo W. Wang Y. Reiser G. (2007). Protease-activated receptors in the brain: receptor expression, activation, and functions in neurodegeneration and neuroprotection. *Brain Res. Rev.* 56 331–345. 10.1016/j.brainresrev.2007.08.002 17915333

[B110] Macchi G. Brahe C. Pomponi M. (1997). Alois Alzheimer and Gaetano Perusini: should man divide what fate united? *Behav. Neurol.* 10 105–108. 10.3233/ben-1997-10401 24486820

[B111] Maggio N. Blatt I. Vlachos A. Tanne D. Chapman J. Segal M. (2013a). Treating seizures and epilepsy with anticoagulants? *Front. Cell Neurosci.* 7:19 10.3389/fncel.2013.00019 PMC3587848 23467310

[B112] Maggio N. Cavaliere C. Papa M. Blatt I. Chapman J. Segal M. (2013b). Thrombin regulation of synaptic transmission: implications for seizure onset. *Neurobiol. Dis.* 50 171–178. 10.1016/j.nbd.2012.10.017 23103417

[B113] Maggio N. Itsekson Z. Dominissini D. Blatt I. Amariglio N. Rechavi G. (2013c). Thrombin regulation of synaptic plasticity: implications for physiology and pathology. *Exp. Neurol.* 247 595–604. 10.1016/j.expneurol.2013.02.011 23454608

[B114] Maggio N. Itsekson Z. Ikenberg B. Strehl A. Vlachos A. Blatt I. (2014). The anticoagulant activated protein C (aPC) promotes metaplasticity in the hippocampus through an EPCR-PAR1-S1P1 receptors dependent mechanism. *Hippocampus* 24 1030–1038. 10.1002/hipo.22288 24753100

[B115] Maggio N. Shavit E. Chapman J. Segal M. (2008). Thrombin induces long-term potentiation of reactivity to afferent stimulation and facilitates epileptic seizures in rat hippocampal slices: toward understanding the functional consequences of cerebrovascular insults. *J. Neurosci.* 28 732–736. 10.1523/jneurosci.3665-07.2008 18199772 PMC6670357

[B116] Maier M. Peng Y. Jiang L. Seabrook T. J. Carroll M. C. Lemere C. A. (2008). Complement C3 deficiency leads to accelerated amyloid beta plaque deposition and neurodegeneration and modulation of the microglia/macrophage phenotype in amyloid precursor protein transgenic mice. *J. Neurosci.* 28 6333–6341. 10.1523/jneurosci.0829-08.2008 18562603 PMC3329761

[B117] Mancuso M. E. Santagostino E. (2017). Platelets: much more than bricks in a breached wall. *Br. J. Haematol.* 178 209–219. 10.1111/bjh.14653 28419428

[B118] Manwell L. A. Barbic S. P. Roberts K. Durisko Z. Lee C. Ware E. (2015). What is mental health? Evidence towards a new definition from a mixed methods multidisciplinary international survey. *BMJ Open* 5:e007079. 10.1136/bmjopen-2014-007079 26038353 PMC4458606

[B119] Markiewski M. M. Lambris J. D. (2009). Unwelcome Complement. *Cancer Res.* 69 6367–6370. 10.1158/0008-5472.CAN-09-1918 19654288 PMC2727567

[B120] Marti-Carvajal A. J. Anand V. Cardona A. F. Sola I. (2014). Eculizumab for treating patients with paroxysmal nocturnal hemoglobinuria. *Cochrane Database Syst. Rev.* 10:Cd010340. 10.1002/14651858.CD010340.pub2 25356860

[B121] Mayeux R. Stern Y. (2012). Epidemiology of Alzheimer disease. *Cold Spring Harb. Perspect Med.* 2:a006239. 10.1101/cshperspect.a006239 22908189 PMC3405821

[B122] Mazarati A. Maroso M. Iori V. Vezzani A. Carli M. (2011). High-mobility group box-1 impairs memory in mice through both toll-like receptor 4 and Receptor for Advanced Glycation End Products. *Exp. Neurol.* 232 143–148. 10.1016/j.expneurol.2011.08.012 21884699 PMC3202022

[B123] Mazza M. Marano G. Traversi G. Bria P. Mazza S. (2011). Primary cerebral blood flow deficiency and Alzheimer’s disease: shadows and lights. *J. Alzheimers. Dis.* 23 375–389. 10.3233/jad-2010-090700 21098977

[B124] McCoy K. L. Gyoneva S. Vellano C. P. Smrcka A. V. Traynelis S. F. Hepler J. R. (2012). Protease-activated receptor 1 (PAR1) coupling to G(q/11) but not to G(i/o) or G(12/13) is mediated by discrete amino acids within the receptor second intracellular loop. *Cell. Signal.* 24 1351–1360. 10.1016/j.cellsig.2012.01.011 22306780 PMC3319227

[B125] McLaughlin J. N. Shen L. Holinstat M. Brooks J. D. Dibenedetto E. Hamm H. E. (2005). Functional selectivity of G protein signaling by agonist peptides and thrombin for the protease-activated receptor-1. *J. Biol. Chem.* 280 25048–25059. 10.1074/jbc.M414090200 15878870

[B126] McMichael M. (2012). New models of hemostasis. *Top. Companion Anim. Med.* 27 40–45. 10.1053/j.tcam.2012.07.005 23031454

[B127] Medzhitov R. Schneider D. S. Soares M. P. (2012). Disease tolerance as a defense strategy. *Science* 335 936–941. 10.1126/science.1214935 22363001 PMC3564547

[B128] Mihara K. Ramachandran R. Saifeddine M. Hansen K. K. Renaux B. Polley D. (2016). Thrombin-mediated direct activation of proteinase-activated receptor-2: another target for thrombin signaling. *Mol. Pharmacol.* 89 606–614. 10.1124/mol.115.102723 26957205

[B129] Mnjoyan Z. Li J. Afshar-Kharghan V. (2008). Factor H binds to platelet integrin alphaIIbbeta3. *Platelets* 19 512–519. 10.1080/09537100802238494 18979363

[B130] Morel A. Miller E. Bijak M. Saluk J. (2016). The increased level of COX-dependent arachidonic acid metabolism in blood platelets from secondary progressive multiple sclerosis patients. *Mol. Cell. Biochem.* 420 85–94. 10.1007/s11010-016-2770-6 27507559 PMC4992022

[B131] Morrell C. N. Aggrey A. A. Chapman L. M. Modjeski K. L. (2014). Emerging roles for platelets as immune and inflammatory cells. *Blood* 123 2759–2767. 10.1182/blood-2013-11-462432 24585776 PMC4007605

[B132] Mosesson M. W. (2005). Fibrinogen and fibrin structure and functions. *J. Thromb. Haemost.* 3 1894–1904. 10.1111/j.1538-7836.2005.01365.x 16102057

[B133] Mosnier L. O. Zlokovic B. V. Griffin J. H. (2014). Cytoprotective-selective activated protein C therapy for ischaemic stroke. *Thromb. Haemost.* 112 883–892. 10.1160/th14-05-0448 25230930 PMC4356129

[B134] Muoio V. Persson P. B. Sendeski M. M. (2014). The neurovascular unit - concept review. *Acta Physiol.* 210 790–798. 10.1111/apha.12250 24629161

[B135] Musselman D. L. Marzec U. Davidoff M. Manatunga A. K. Gao F. Reemsnyder A. (2002). Platelet activation and secretion in patients with major depression, thoracic aortic atherosclerosis, or renal dialysis treatment. *Depress. Anxiety* 15 91–101. 10.1002/da.10020 12001177

[B136] Musselman D. L. Tomer A. Manatunga A. K. Knight B. T. Porter M. R. Kasey S. (1996). Exaggerated platelet reactivity in major depression. *Am. J. Psychiatry* 153 1313–1317. 10.1176/ajp.153.10.1313 8831440

[B137] Muszbek L. Bereczky Z. Bagoly Z. Komaromi I. Katona E. (2011). Factor XIII: a coagulation factor with multiple plasmatic and cellular functions. *Physiol. Rev.* 91 931–972. 10.1152/physrev.00016.2010 21742792

[B138] Nagai T. Nabeshima T. Yamada K. (2008). Basic and translational research on proteinase-activated receptors: regulation of nicotine reward by the tissue plasminogen activator (tPA) - plasmin system via proteinase-activated receptor 1. *J. Pharmacol. Sci.* 108 408–414. 10.1254/jphs.08R04FM 19098386

[B139] Nemeroff C. B. Musselman D. L. (2000). Are platelets the link between depression and ischemic heart disease? *Am. Heart J*. 140(4 Suppl.), 57–62. 11011349 10.1067/mhj.2000.109978

[B140] Nguyen V. A. Carey L. M. Giummarra L. Faou P. Cooke I. Howells D. W. (2016). A pathway proteomic profile of ischemic stroke survivors reveals innate immune dysfunction in association with mild symptoms of depression – a pilot study. *Front. Neurol.* 7:85. 10.3389/fneur.2016.00085 27379006 PMC4907034

[B141] Nicole O. Goldshmidt A. Hamill C. E. Sorensen S. D. Sastre A. Lyuboslavsky P. (2005). Activation of protease-activated receptor-1 triggers astrogliosis after brain injury. *J. Neurosci.* 25 4319–4329. 10.1523/jneurosci.5200-04.2005 15858058 PMC6725104

[B142] Niego B. E. Samson A. L. Petersen K.-U. Medcalf R. L. (2011). Thrombin-induced activation of astrocytes in mixed rat hippocampal cultures is inhibited by soluble thrombomodulin. *Brain Res.* 1381 38–51. 10.1016/j.brainres.2011.01.016 21241677

[B143] Nurden A. T. (2011). Platelets, inflammation and tissue regeneration. *Thromb. Haemost.* 105(Suppl. 1), S13–S33. 10.1160/ths10-11-0720 21479340

[B144] O’Brien C. P. (2011). Evidence-Based Treatments of Addiction. *FOCUS* 9 107–117. 10.1176/foc.9.1.foc107

[B145] Olianas M. C. Dedoni S. Onali P. (2007). Proteinase-activated receptors 1 and 2 in rat olfactory system: layer-specific regulation of multiple signaling pathways in the main olfactory bulb and induction of neurite retraction in olfactory sensory neurons. *Neuroscience* 146 1289–1301. 10.1016/j.neuroscience.2007.02.059 17434682

[B146] Osborn T. M. Dahlgren C. Hartwig J. H. Stossel T. P. (2007). Modifications of cellular responses to lysophosphatidic acid and platelet-activating factor by plasma gelsolin. *Am. J. Physiol. Cell Physiol.* 292 C1323–C1330. 10.1152/ajpcell.00510.2006 17135294

[B147] Ossovskaya V. S. Bunnett N. W. (2004). Protease-activated receptors: contribution to physiology and disease. *Physiol. Rev.* 84 579–621. 10.1152/physrev.00028.2003 15044683

[B148] Pang P. T. Teng H. K. Zaitsev E. Woo N. T. Sakata K. Zhen S. (2004). Cleavage of proBDNF by tPA/plasmin is essential for long-term hippocampal plasticity. *Science* 306 487–491. 10.1126/science.1100135 15486301

[B149] Papa M. De Luca C. Petta F. Alberghina L. Cirillo G. (2014). Astrocyte-neuron interplay in maladaptive plasticity. *Neurosci. Biobehav. Rev.* 42 35–54. 10.1016/j.neubiorev.2014.01.010 24509064

[B150] Parissis J. T. Fountoulaki K. Filippatos G. Adamopoulos S. Paraskevaidis I. Kremastinos D. (2007). Depression in coronary artery disease: novel pathophysiologic mechanisms and therapeutic implications. *Int. J. Cardiol.* 116 153–160. 10.1016/j.ijcard.2006.03.038 16822560

[B151] Pasinetti G. M. Johnson S. A. Rozovsky I. Lampert-Etchells M. Morgan D. G. Gordon M. N. (1992). Complement C1qB and C4 mRNAs responses to lesioning in rat brain. *Exp. Neurol.* 118 117–125. 10.1016/0014-4886(92)90028-O 1426121

[B152] Pawlak R. Melchor J. P. Matys T. Skrzypiec A. E. Strickland S. (2005). Ethanol-withdrawal seizures are controlled by tissue plasminogen activator via modulation of NR2B-containing NMDA receptors. *Proc. Natl. Acad. Sci. U.S.A.* 102 443–448. 10.1073/pnas.0406454102 15630096 PMC544297

[B153] Pedersen E. D. Waje-Andreassen U. Vedeler C. A. Aamodt G. Mollnes T. E. (2004). Systemic complement activation following human acute ischaemic stroke. *Clin. Exp. Immunol.* 137 117–122. 10.1111/j.1365-2249.2004.02489.x 15196251 PMC1809093

[B154] Peerbooms O. L. van Os J. Drukker M. Kenis G. Hoogveld L. de Hert M. (2011). Meta-analysis of MTHFR gene variants in schizophrenia, bipolar disorder and unipolar depressive disorder: evidence for a common genetic vulnerability? *Brain Behav. Immun.* 25 1530–1543. 10.1016/j.bbi.2010.12.006 21185933

[B155] Peraramelli S. Rosing J. Hackeng T. M. (2012). TFPI-dependent activities of protein S. *Thromb. Res.* 129(Suppl. 2), S23–S26. 10.1016/j.thromres.2012.02.024 22425215

[B156] Popova S. N. Bergqvist M. Dimberg A. Edqvist P. H. Ekman S. Hesselager G. (2014). Subtyping of gliomas of various WHO grades by the application of immunohistochemistry. *Histopathology* 64 365–379. 10.1111/his.12252 24410805 PMC4670475

[B157] Procaccianti G. Zaniboni A. Rondelli F. Crisci M. Sacquegna T. (2012). Seizures in acute stroke: incidence, risk factors and prognosis. *Neuroepidemiology* 39 45–50. 10.1159/000338374 22777596

[B158] Qin C. Fan W. H. Liu Q. Shang K. Murugan M. Wu L. J. (2017). fingolimod protects against ischemic white matter damage by modulating microglia toward M2 polarization via STAT3 pathway. *Stroke* 48 3336–3346. 10.1161/strokeaha.117.018505 29114096 PMC5728178

[B159] Rajput P. S. Lyden P. D. Chen B. Lamb J. A. Pereira B. Lamb A. (2014). Protease activated receptor-1 mediates cytotoxicity during ischemia using in vivo and in vitro models. *Neuroscience* 281 229–240. 10.1016/j.neuroscience.2014.09.038 25261684 PMC4377119

[B160] Ramanathan A. Nelson A. R. Sagare A. P. Zlokovic B. V. (2015). Impaired vascular-mediated clearance of brain amyloid beta in Alzheimer’s disease: the role, regulation and restoration of LRP1. *Front. Aging Neurosci.* 7:136 10.3389/fnagi.2015.00136 PMC4502358 26236233

[B161] Raphael I. Webb J. Stuve O. Haskins W. Forsthuber T. (2015). Body fluid biomarkers in multiple sclerosis: how far we have come and how they could affect the clinic now and in the future. *Expert Rev. Clin. Immunol.* 11 69–91. 10.1586/1744666x.2015.991315 25523168 PMC4326231

[B162] Reiman E. M. (2014). Alzheimer’s disease and other dementias: advances in 2013. *Lancet Neurol.* 13 3–5. 10.1016/s1474-4422(13)70257-6 24331781

[B163] Reitz C. Brayne C. Mayeux R. (2011). Epidemiology of Alzheimer disease. *Nat. Rev. Neurol.* 7 137–152. 10.1038/nrneurol.2011.2 21304480 PMC3339565

[B164] Riewald M. Ruf W. (2005). Protease-activated receptor-1 signaling by activated protein C in cytokine-perturbed endothelial cells is distinct from thrombin signaling. *J. Biol. Chem.* 280 19808–19814. 10.1074/jbc.M500747200 15769747

[B165] Roostaei T. Sadaghiani S. Mashhadi R. Falahatian M. Mohamadi E. Javadian N. (2018). Convergent effects of a functional C3 variant on brain atrophy, demyelination, and cognitive impairment in multiple sclerosis. *Mult Scler* 10.1177/1352458518760715 [Epub ahead of print]. 29485352

[B166] Russo A. Soh U. J. Paing M. M. Arora P. Trejo J. (2009). Caveolae are required for protease-selective signaling by protease-activated receptor-1. *Proc. Natl. Acad. Sci. U.S.A.* 106 6393–6397. 10.1073/pnas.0810687106 19332793 PMC2669384

[B167] Ryu J. K. Petersen M. A. Murray S. G. Baeten K. M. Meyer-Franke A. Chan J. P. (2015). Blood coagulation protein fibrinogen promotes autoimmunity and demyelination via chemokine release and antigen presentation. *Nat. Commun.* 6:8164. 10.1038/ncomms9164 26353940 PMC4579523

[B168] Salminen A. Ojala J. Kauppinen A. Kaarniranta K. Suuronen T. (2009). Inflammation in Alzheimer’s disease: Amyloid-β oligomers trigger innate immunity defence via pattern recognition receptors. *Prog. Neurobiol.* 87 181–194. 10.1016/j.pneurobio.2009.01.001 19388207

[B169] Sanchez-Mejia R. O. Mucke L. (2010). Phospholipase A2 and arachidonic acid in Alzheimer’s disease. *Biochim. Biophys. Acta* 1801 784–790. 10.1016/j.bbalip.2010.05.013 20553961 PMC3024142

[B170] Sarangi P. P. Lee H. W. Kim M. (2010). Activated protein C action in inflammation. *Br. J. Haematol.* 148 817–833. 10.1111/j.1365-2141.2009.08020.x 19995397 PMC2868910

[B171] Sawcer S. Franklin R. J. Ban M. (2014). Multiple sclerosis genetics. *Lancet Neurol.* 13 700–709. 10.1016/s1474-4422(14)70041-9 24852507

[B172] Schafer M. K. Schwaeble W. J. Post C. Salvati P. Calabresi M. Sim R. B. (2000). Complement C1q is dramatically up-regulated in brain microglia in response to transient global cerebral ischemia. *J. Immunol.* 164 5446–5452. 10.4049/jimmunol.164.10.5446 10799911

[B173] Schneider J. A. (2016). The cerebral cortex in cerebral amyloid angiopathy. *Lancet Neurol.* 15 778–779. 10.1016/s1474-4422(16)30100-4 27302344 PMC13747226

[B174] Schroeder V. Borner U. Gutknecht S. Schmid J. P. Saner H. Kohler H. P. (2007). Relation of depression to various markers of coagulation and fibrinolysis in patients with and without coronary artery disease. *Eur. J. Cardiovasc. Prev. Rehabil.* 14 782–787. 10.1097/HJR.0b013e32828622e8 18043299

[B175] Schultz S. J. Aly H. Hasanen B. M. Khashaba M. T. Lear S. C. Bendon R. W. (2005). Complement component 9 activation, consumption, and neuronal deposition in the post-hypoxic-ischemic central nervous system of human newborn infants. *Neurosci. Lett.* 378 1–6. 10.1016/j.neulet.2004.12.008 15763162

[B176] Schwab C. Klegeris A. McGeer P. L. (2010). Inflammation in transgenic mouse models of neurodegenerative disorders. *Biochim. Biophys. Acta* 1802 889–902. 10.1016/j.bbadis.2009.10.013 19883753

[B177] Sen S. Nesse R. M. Stoltenberg S. F. Li S. Gleiberman L. Chakravarti A. (2003). A BDNF coding variant is associated with the NEO personality inventory domain neuroticism, a risk factor for depression. *Neuropsychopharmacology* 28 397–401. 10.1038/sj.npp.1300053 12589394

[B178] Shavit Stein E. Ben Shimon M. Artan Furman A. Golderman V. Chapman J. Maggio N. (2018). Thrombin Inhibition Reduces the Expression of Brain Inflammation Markers upon Systemic LPS Treatment. *Neural Plast.* 2018:7692182. 10.1155/2018/7692182 30018633 PMC6029482

[B179] Siegenthaler B. Bali J. Rajendran L. (2016). γ-Secretase regulates the α-secretase cleavage of the Alzheimer’s disease, amyloid precursor protein. *Matters*. 10.19185/matters.201601000003

[B180] Skovronsky D. M. Lee V. M. Pratico D. (2001). Amyloid precursor protein and amyloid beta peptide in human platelets. Role of cyclooxygenase and protein kinase C. *J. Biol. Chem.* 276 17036–17043. 10.1074/jbc.M006285200 11278299

[B181] Soh U. J. Trejo J. (2011). Activated protein C promotes protease-activated receptor-1 cytoprotective signaling through beta-arrestin and dishevelled-2 scaffolds. *Proc. Natl. Acad. Sci. U.S.A.* 108 E1372–E1380. 10.1073/pnas.1112482108 22106258 PMC3250136

[B182] Sotnikov I. Veremeyko T. Starossom S. C. Barteneva N. Weiner H. L. Ponomarev E. D. (2013). Platelets recognize brain-specific glycolipid structures, respond to neurovascular damage and promote neuroinflammation. *PLoS One* 8:e58979. 10.1371/journal.pone.0058979 23555611 PMC3608633

[B183] Spivak B. Radwan M. Brandon J. Baruch Y. Stawski M. Tyano S. (1993). Reduced total complement haemolytic activity in schizophrenic patients. *Psychol. Med.* 23 315–318. 10.1017/S0033291700028397 8332647

[B184] Starossom S. C. Veremeyko T. Yung A. W. Dukhinova M. Au C. Lau A. Y. (2015). Platelets play differential role during the initiation and progression of autoimmune neuroinflammation. *Circ. Res.* 117 779–792. 10.1161/circresaha.115.306847 26294656 PMC4716010

[B185] Stein E. S. Itsekson-Hayosh Z. Aronovich A. Reisner Y. Bushi D. Pick C. G. (2015). Thrombin induces ischemic LTP (iLTP): implications for synaptic plasticity in the acute phase of ischemic stroke. *Sci. Rep.* 5:7912. 10.1038/srep07912 25604482 PMC4300504

[B186] Stevens B. Allen N. J. Vazquez L. E. Howell G. R. Christopherson K. S. Nouri N. (2007). The classical complement cascade mediates CNS synapse elimination. *Cell* 131 1164–1178. 10.1016/j.cell.2007.10.036 18083105

[B187] Stolz L. Derouiche A. Devraj K. Weber F. Brunkhorst R. Foerch C. (2017). Anticoagulation with warfarin and rivaroxaban ameliorates experimental autoimmune encephalomyelitis. *J. Neuroinflammation* 14:152. 10.1186/s12974-017-0926-2 28754118 PMC5534067

[B188] Strenn N. Suchankova P. Nilsson S. Fischer C. Wegener G. Mathe A. A. (2015). Expression of inflammatory markers in a genetic rodent model of depression. *Behav. Brain Res.* 281 348–357. 10.1016/j.bbr.2014.09.025 25277840

[B189] Sun N. Shen Y. Han W. Shi K. Wood K. Fu Y. (2016). Selective sphingosine-1-phosphate receptor 1 modulation attenuates experimental intracerebral hemorrhage. *Stroke* 47 1899–1906. 10.1161/strokeaha.115.012236 27174529

[B190] Suzuki-Inoue K. Kato Y. Inoue O. Kaneko M. K. Mishima K. Yatomi Y. (2007). Involvement of the snake toxin receptor CLEC-2, in podoplanin-mediated platelet activation, by cancer cells. *J. Biol. Chem.* 282 25993–26001. 10.1074/jbc.M702327200 17616532

[B191] Szaflarski J. P. Rackley A. Y. Kleindorfer D. O. Khoury J. Woo D. Miller R. (2008). Incidence of seizures in the acute phase of stroke: a population-based study. *Epilepsia* 49 974–981. 10.1111/j.1528-1167.2007.01513.x 18248443 PMC5316476

[B192] Tanaka K. A. Key N. S. Levy J. H. (2009). Blood coagulation: hemostasis and thrombin regulation. *Anesth. Analg.* 108 1433–1446. 10.1213/ane.0b013e31819bcc9c 19372317

[B193] Ten V. S. Sosunov S. A. Mazer S. P. Stark R. I. Caspersen C. Sughrue M. E. (2005). C1q-deficiency is neuroprotective against hypoxic-ischemic brain injury in neonatal mice. *Stroke* 36 2244–2250. 10.1161/01.STR.0000182237.20807.d0 16179576

[B194] Thal D. R. Griffin W. S. de Vos R. A. Ghebremedhin E. (2008). Cerebral amyloid angiopathy and its relationship to Alzheimer’s disease. *Acta Neuropathol.* 115 599–609. 10.1007/s00401-008-0366-2 18369648

[B195] Theodorou G. L. Marousi S. Ellul J. Mougiou A. Theodori E. Mouzaki A. (2008). T helper 1 (Th1)/Th2 cytokine expression shift of peripheral blood CD4+ and CD8+ T cells in patients at the post-acute phase of stroke. *Clin. Exp. Immunol.* 152 456–463. 10.1111/j.1365-2249.2008.03650.x 18422734 PMC2453204

[B196] Thombs B. D. Bass E. B. Ford D. E. Stewart K. J. Tsilidis K. K. Patel U. (2006). Prevalence of depression in survivors of acute myocardial infarction. *J. Gen. Intern. Med.* 21 30–38. 10.1111/j.1525-1497.2005.00269.x 16423120 PMC1484630

[B197] Thornton P. McColl B. W. Greenhalgh A. Denes A. Allan S. M. Rothwell N. J. (2010). Platelet interleukin-1alpha drives cerebrovascular inflammation. *Blood* 115 3632–3639. 10.1182/blood-2009-11-252643 20200351

[B198] Traynelis S. F. Trejo J. (2007). Protease-activated receptor signaling: new roles and regulatory mechanisms. *Curr. Opin. Hematol* 14 230–235. 10.1097/MOH.0b013e3280dce568 17414212

[B199] Trebst C. Jarius S. Berthele A. Paul F. Schippling S. Wildemann B. (2014). Update on the diagnosis and treatment of neuromyelitis optica: recommendations of the Neuromyelitis Optica Study Group (NEMOS). *J. Neurol.* 261 1–16. 10.1007/s00415-013-7169-7 24272588 PMC3895189

[B200] Tsilibary E. Tzinia A. Radenovic L. Stamenkovic V. Lebitko T. Mucha M. (2014). Neural ECM proteases in learning and synaptic plasticity. *Prog. Brain Res.* 214 135–157. 10.1016/b978-0-444-63486-3.00006-2 25410356

[B201] Tsivgoulis G. Katsanos A. H. Alexandrov A. V. (2014). Reperfusion therapies of acute ischemic stroke: potentials and failures. *Front. Neurol.* 5:215. 10.3389/fneur.2014.00215 25404927 PMC4217479

[B202] van Nispen tot Pannerden H. de Haas F. Geerts W. Posthuma G. van Dijk S. Heijnen H. F. (2010). The platelet interior revisited: electron tomography reveals tubular alpha-granule subtypes. *Blood* 116 1147–1156. 10.1182/blood-2010-02-268680 20439620

[B203] Vance K. M. Rogers R. C. Hermann G. E. (2015). PAR1-activated astrocytes in the nucleus of the solitary tract stimulate adjacent neurons via NMDA receptors. *J. Neurosci.* 35 776–785. 10.1523/jneurosci.3105-14.2015 25589770 PMC4293422

[B204] Vasthare U. S. Barone F. C. Sarau H. M. Rosenwasser R. H. DiMartino M. Young W. F. (1998). Complement depletion improves neurological function in cerebral ischemia. *Brain Res. Bull.* 45 413–419. 10.1016/S0361-9230(97)00408-5 9527016

[B205] Vaughan P. J. Su J. Cotman C. W. Cunningham D. D. (1994). Protease nexin-1, a potent thrombin inhibitor, is reduced around cerebral blood vessels in Alzheimer’s disease. *Brain Res.* 668 160–170. 10.1016/0006-8993(94)90521-5 7704602

[B206] Voss B. McLaughlin J. N. Holinstat M. Zent R. Hamm H. E. (2007). PAR1, but not PAR4, activates human platelets through a Gi/o/phosphoinositide-3 kinase signaling axis. *Mol. Pharmacol.* 71 1399–1406. 10.1124/mol.106.033365 17303701

[B207] Walter J. Handel L. L. Brodhun M. van Rossum D. Hanisch U. K. Liebmann L. (2012). Expression of coagulation factors and their receptors in tumor tissue and coagulation factor upregulation in peripheral blood of patients with cerebral carcinoma metastases. *J. Cancer Res. Clin. Oncol.* 138 141–151. 10.1007/s00432-011-1078-x 22065054 PMC11824165

[B208] Wang H. Reiser G. (2003). Thrombin signaling in the brain: the role of protease-activated receptors. *Biol. Chem.* 384 193–202. 10.1515/bc.2003.021 12675511

[B209] Wang H. Ricklin D. Lambris J. D. (2017). Complement-activation fragment C4a mediates effector functions by binding as untethered agonist to protease-activated receptors 1 and 4. *Proc. Natl. Acad. Sci. U.S.A.* 114 10948–10953. 10.1073/pnas.1707364114 28973891 PMC5642699

[B210] Wang H. Y. Markowitz P. Levinson D. Undie A. S. Friedman E. (1999). Increased membrane-associated protein kinase C activity and translocation in blood platelets from bipolar affective disorder patients. *J. Psychiatr. Res.* 33 171–179. 10.1016/S0022-3956(98)90057-7 10221749

[B211] Wang Y. Richter-Landsberg C. Reiser G. (2004). Expression of protease-activated receptors (PARs) in OLN-93 oligodendroglial cells and mechanism of PAR-1-induced calcium signaling. *Neuroscience* 126 69–82. 10.1016/j.neuroscience.2004.03.024 15145074

[B212] Wiesner T. Bugl S. Mayer F. Hartmann J. T. Kopp H. G. (2010). Differential changes in platelet VEGF, Tsp, CXCL12, and CXCL4 in patients with metastatic cancer. *Clin. Exp. Metastasis* 27 141–149. 10.1007/s10585-010-9311-6 20182908

[B213] Wittinghofer A. Vetter I. R. (2011). Structure-function relationships of the G domain, a canonical switch motif. *Annu. Rev. Biochem.* 80 943–971. 10.1146/annurev-biochem-062708-134043 21675921

[B214] Wojtukiewicz M. Z. Hempel D. Sierko E. Tucker S. C. Honn K. V. (2016). Thrombin—unique coagulation system protein with multifaceted impacts on cancer and metastasis. *Cancer Metastasis Rev.* 35 213–233. 10.1007/s10555-016-9626-0 27189210

[B215] Wyss-Coray T. Yan F. Lin A. H. Lambris J. D. Alexander J. J. Quigg R. J. (2002). Prominent neurodegeneration and increased plaque formation in complement-inhibited Alzheimer’s mice. *Proc. Natl. Acad. Sci. U.S.A.* 99 10837–10842. 10.1073/pnas.162350199 12119423 PMC125059

[B216] Xi G. Hua Y. Keep R. F. Younger J. G. Hoff J. T. (2001). Systemic complement depletion diminishes perihematomal brain edema in rats. *Stroke* 32 162–167. 10.1161/01.STR.32.1.162 11136932

[B217] Yan W. Che L. Jiang J. Yang F. Duan Q. Song H. (2016). Depletion of complement system immunity in patients with myocardial infarction. *Mol. Med. Rep.* 14 5350–5356. 10.3892/mmr.2016.5912 27840920

[B218] Ye J. Rezaie A. R. Esmon C. T. (1994). Glycosaminoglycan contributions to both protein C activation and thrombin inhibition involve a common arginine-rich site in thrombin that includes residues arginine 93, 97, and 101. *J. Biol. Chem.* 269 17965–17970. 8027055

[B219] Zamolodchikov D. Renne T. Strickland S. (2016). The Alzheimer’s disease peptide beta-amyloid promotes thrombin generation through activation of coagulation factor XII. *J. Thromb. Haemost.* 14 995–1007. 10.1111/jth.13209 26613657 PMC4870142

[B220] Zaslavsky A. Baek K. H. Lynch R. C. Short S. Grillo J. Folkman J. (2010). Platelet-derived thrombospondin-1 is a critical negative regulator and potential biomarker of angiogenesis. *Blood* 115 4605–4613. 10.1182/blood-2009-09-242065 20086246 PMC2881490

[B221] Zhang Y. Zhan H. Xu W. Yuan Z. Lu P. Zhan L. (2011). Upregulation of matrix metalloproteinase-1 and proteinase-activated receptor-1 promotes the progression of human gliomas. *Pathol. Res. Pract.* 207 24–29. 10.1016/j.prp.2010.10.003 21087829

[B222] Zimmer G. Schanuel S. M. Burger S. Weth F. Steinecke A. Bolz J. (2010). Chondroitin sulfate acts in concert with semaphorin 3A to guide tangential migration of cortical interneurons in the ventral telencephalon. *Cereb. Cortex* 20 2411–2422. 10.1093/cercor/bhp309 20071458

[B223] Zlotnik A. Yoshie O. (2000). Chemokines: a new classification system and their role in immunity. *Immunity* 12 121–127. 10.1016/S1074-7613(00)80165-X 10714678

[B224] Zlotnik A. Yoshie O. (2012). The chemokine superfamily revisited. *Immunity* 36 705–716. 10.1016/j.immuni.2012.05.008 22633458 PMC3396424

